# Engineering blood exosomes for tumor-targeting efficient gene/chemo combination therapy

**DOI:** 10.7150/thno.45028

**Published:** 2020-06-22

**Authors:** Qi Zhan, Kaikai Yi, Hongzhao Qi, Sidi Li, Xueping Li, Qixue Wang, Yunfei Wang, Chaoyong Liu, Mingzhe Qiu, Xubo Yuan, Jin Zhao, Xin Hou, Chunsheng Kang

**Affiliations:** 1 Tianjin Key Laboratory of Composite and Functional Materials, School of Material Science and Engineering, Tianjin University, Tianjin 300072, China.; 2 Department of Neurosurgery, Tianjin Medical University General Hospital, Laboratory of Neuro-oncology, Tianjin Neurological Institute, Key Laboratory of Post-Neurotrauma Neuro-Repair and Regeneration in Central Nervous System, Ministry of Education and Tianjin City, Tianjin 300052, China.; 3 Institute for Translational Medicine, Qingdao University, Qingdao, 266021, China.

**Keywords:** exosome, co-loading, tumor targeting, efficient transfection, combination therapy

## Abstract

**Rationale:** Developing an effective nanoplatform to realize 'multi-in-one' is essential to broaden the therapeutic potential of combination therapy. Exosomes are ideal candidates since their intrinsic abilities of integrating multiple contents and functions. However, only limited efforts have been devoted to engineering exosomes to integrate the needed properties, also considering the safety and yield, for tumor-targeted and efficient gene/chemo combination therapy.

**Methods:** Herein, by manipulating the exosome membrane, blood exosomes with high abundance and safety are engineered as a versatile combinatorial delivery system, where the doxorubicin (Dox) and cholesterol-modified miRNA21 inhibitor (miR-21i) are co-embedded into the lipid bilayer of exosomes, and the magnetic molecules and endosomolytic peptides L17E are bind to the exosome membrane through ligand-receptor coupling and electrostatic interactions, respectively.

**Results:** It is proved that such engineering strategy not only preserves their intrinsic features, but also readily integrates multiple properties of tumor targeting, efficient transfection and gene/chemo combination therapy into blood exosomes. The lipid bilayer structure of exosomes allows them to co-load Dox and miR-21i with high-payloads. Moreover, profiting from the integration of magnetic molecules and L17E peptides, the engineered exosomes exhibit an enhanced tumor accumulation and an improved endosome escape ability, thereby specifically and efficiently delivering encapsulated cargos to tumor cells. As a result, a remarkable inhibition of tumor growth is observed in the tumor-bearing mice, and without noticeable side effects.

**Conclusions:** This study demonstrates the potential of engineered blood exosomes as feasible co-delivery nanosystem for tumor-targeted and efficient combination therapy. Further development by replacing the drugs combined regimens can potentially make this engineered exosome become a general platform for the design of safe and effective combination therapy modality.

## Introduction

Over the last years, nanomedicine that integrates chemotherapy and gene therapy in a single nanoplatform has been extensively proved as a significant paradigm for cancer treatment 1-3. The key for the design of such nanomedicine is the integration of required components, each with different functions, into one nanosystem 4-6. A widely-used integration strategy is based on various material synthesis methods, designing the nanoparticles as a core-shell structure to co-load two therapeutic agents 7, 8, and introducing multiple functional molecules, such as polyethylene glycol (PEG) 9, 10, targeting ligands 11, 12 and cationic charges 13, 14, to maximize its delivery efficiency. Moreover, some stimuli-responsive 15-18 or multistage nanosystems 19-22 have tried to integrate the functions of various materials, with all components working in a coordinated way, thus enabling them as nanomedicines with high combination therapeutic efficacy. Although these are fantastic works, more efforts have to be dedicated to explore a high-performance and clinically available nanoplatform capable of co-delivering drugs and nucleic acids, and with optimized or additional properties. Natural intercellular delivery vectors, such as exosomes, which can transfer several cargos once in all 23, theoretically provide an ideal nanoplatform for the development of new integration strategies.

Exosomes, including natural 'Trojan Horses', are being transformed to the next-generation theranostic nanomedicine 24, 25. Clinical trials on exosome-based therapies have produced enticing results and are rapidly increasing 26, 27. Exosomes are nanoscale vesicles released by various cell types as well as present in almost all body fluids, and naturally possess the properties of integrating multiple contents and physiological functions 28, 29. Exosomes, composed of lipid bilayer enclosed nano-sized extracellular vesicles, contain coding and noncoding RNAs 30, 31, and provide loading sites for the introduction of hydrophobic drugs. Moreover, the ability of exosomes to negotiate complex *in vivo* delivery hurdles, including monocyte clearance, cell adhesion and endocytosis, is attributed to the multivalent integration of specific proteins (e.g. CD47, CD63 and CD9) on their membrane, and its diversity and intricacy are difficult to replicate in synthetic nanosystems 24, 32, 33. Given this inherent integration as well as their more attractive stability and long-circulation feature than any other nanocarriers 34-36, it is reasonable to envisage the application of exosomes as new nanoplatform for gene/chemo combination therapy. There are seldom reports on the use of exosomes as co-delivery vehicles 37, which are simply based on their intrinsic nanoscale and blood circulation properties. However, the essential integration nature of exosomes described above has not received sufficient attention, development and expansion in current strategies. The development of engineered exosomes capable of integrating multiple functional components for tumor-targeted and efficient gene/chemo combined therapy is still an unsolved problem to date. Compared with *in vitro* source, blood exosomes mainly secreted by reticulocytes (RTC) are a potential source of safe and sufficient exosomes, as they integrate various membrane proteins including transferrin (Tf) receptors but without any immune- and cancer-stimulating activities 38. It is, therefore, necessary to develop a novel and practical strategy to engineer blood exosomes for combination therapy, which not only realize the co-loading of chemotherapeutants (mostly hydrophobic drugs) and nucleic acids, and more importantly, the introduction of functional moieties to optimize the tumor-targeting and endosome escaping.

**Scheme 1:**
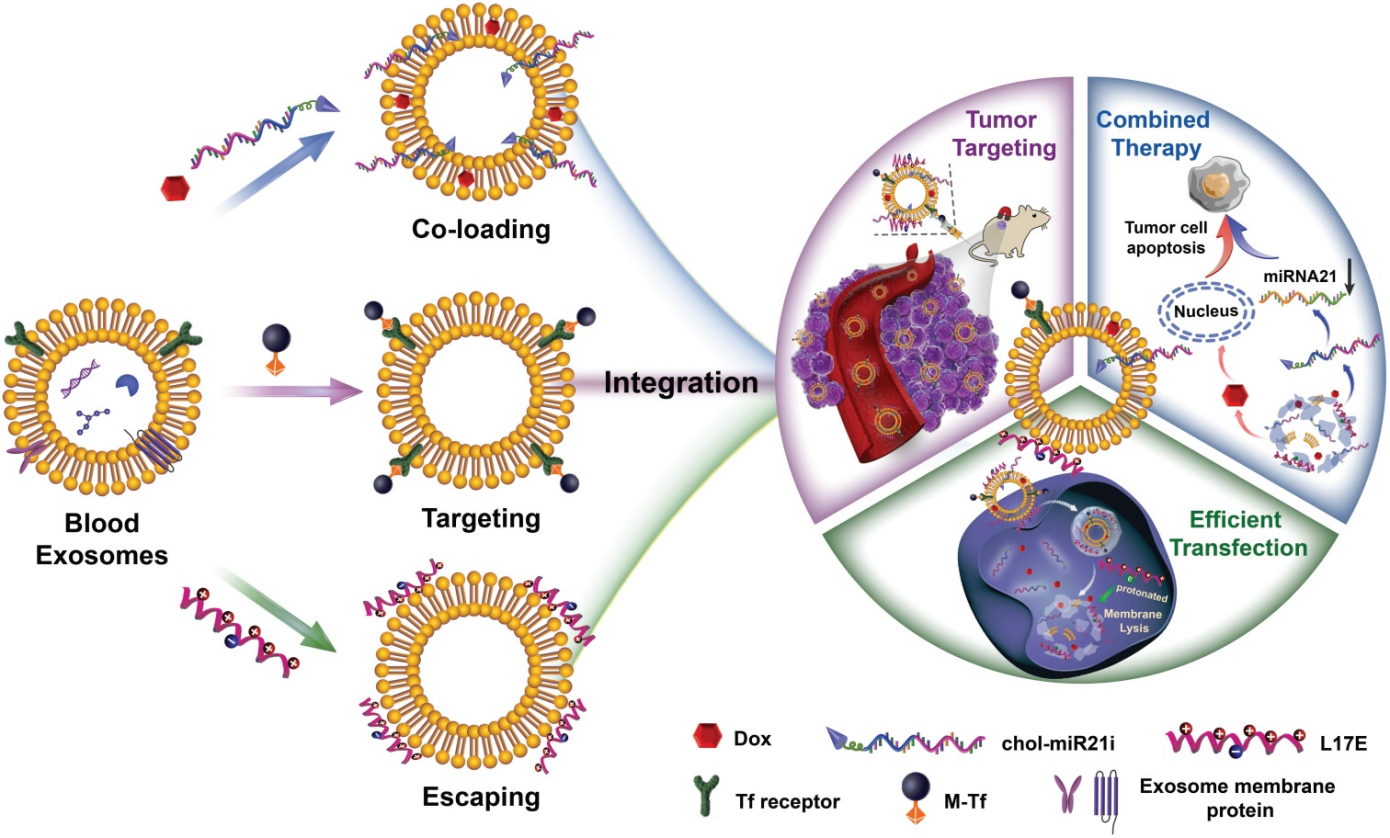
** Schematic representation of engineered blood exosomes for effective gene/chemo combined antitumor therapy.** Taking full use of the structure and properties of the exosome membrane, such blood exosome-based co-delivery nanosystem tactfully integrates three moieties: co-loading of drugs and nucleic acids with high-payloads, tumor targeting and endosomal escaping. After intravenous injection, the engineered exosomes can accumulate at the tumor site with high-efficiency under an external magnetic field, following cellular uptake, the presence of endosomolytic peptide facilitates the effective release of therapeutic RNAs and chemotherapeutic drugs, resulting in a significantly improved gene/chemo combination antitumor effect.

Herein, we explored the novel concept of engineering blood exosomes as co-delivery nanosystems, which integrate three extraordinary functions: flexible and efficient co-loading of drugs and nucleic acids, tumor targeting and endosomal escaping. Specifically, as shown in Scheme 1, taking full use of the structure and biochemical composition of exosomal membrane, this integration was effectuated by a three-part membrane decoration strategy: i) binding ligand-coupled superparamagnetic nanoparticles to the specific membrane proteins of exosome to achieve the separation, purification and tumor magnetic-targeting of exosome; ii) incorporating hydrophobic drugs and hydrophobically modified RNAs into the hydrophobic regions of exosomal membrane for carrying out co-loading; iii) absorbing cationic endosomolytic peptides onto the negatively-charged membrane surface of exosome to promote the cytosolic release of encapsulated cargos. Based on this strategy, the blood exosome-based superparamagnetic nanoparticle cluster was first constructed according to our previously reported method 39, thereby introducing tumor-targeting functions into exosomes. Then, the chemotherapy drug doxorubicin (Dox) and cholesterol-modified single-stranded miRNA21 inhibitor (chol-miR21i) were assembled onto exosome to achieve the integration of two anticancer modalities into one nanoplatform. Furthermore, a cationic lipid-sensitive endosomolytic peptide, L17E peptide 40, was introduced into this exosome-based co-delivery system as the components that promoted cytosolic release of cargos, especially RNAs. We demonstrated that this blood exosome-based nanosystem is able to integrate three functions we designed, thus co-loading of Dox and chol-miR21i into one exosome and co-delivering them into tumor cells with superior tumor accumulation improved cytosolic release. These efficiently released drugs and RNAs simultaneously interfere with nuclear DNA activity and down-regulate the expression of oncogenes, thus remarkably inhibiting the growth of the tumors and alleviating side effects.

## Results and Discussion

### Design, construction and characterization of D-Exos/miR21i-L17E

**Figure 1:**
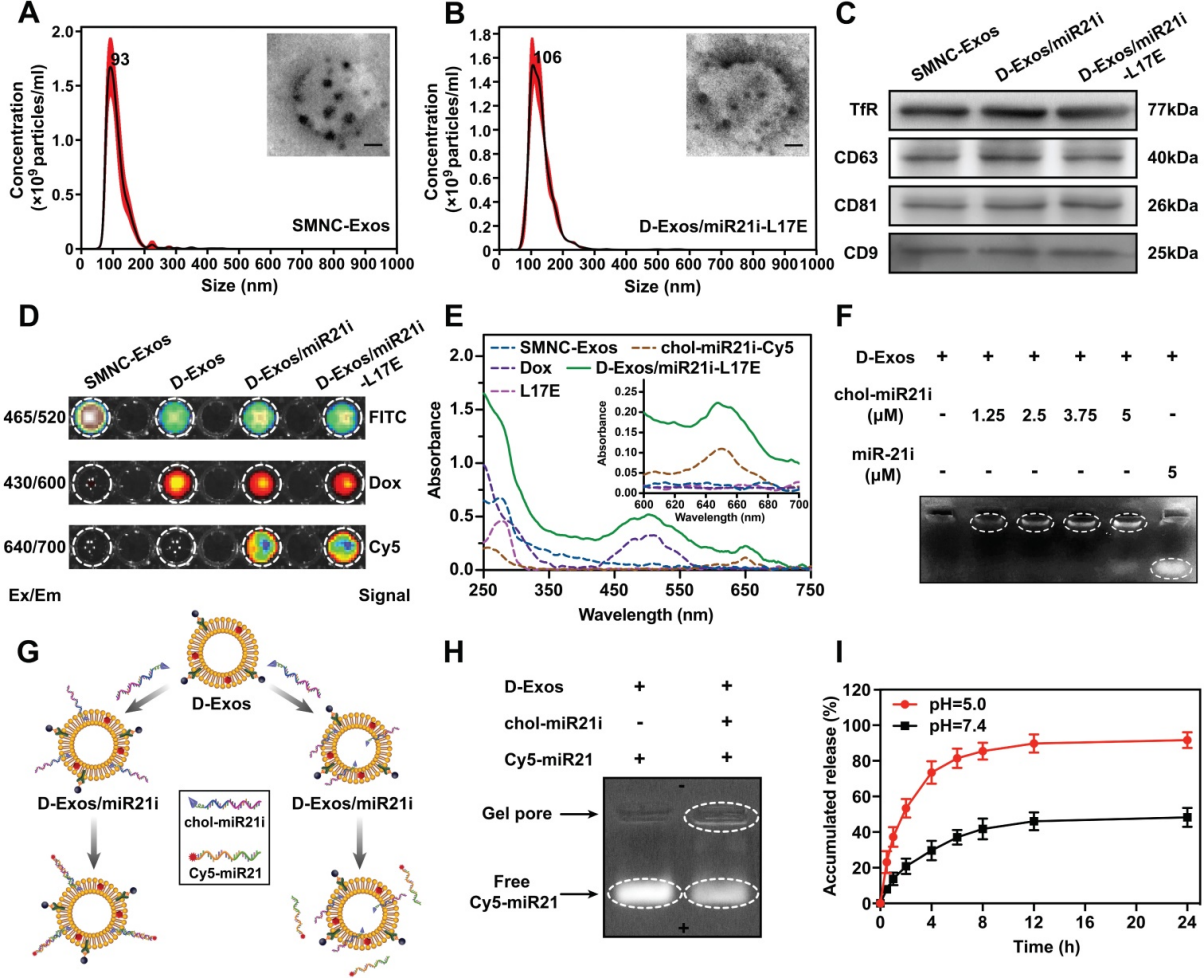
** Characterization of D-Exos/miR21i-L17E.** (A, B) The particle size distribution and representative TEM images of SMNC-Exos and D-Exos/miR21i-L17E, respectively. (scale bar: 20 nm) (C) Western blots analysis of specific Tf receptors and exosome marker proteins (CD63, CD81 and CD9) of SMNC-Exos, D-Exos/miR21i and D-Exos/miR21i-L17E. (D) Detection of fluorescent signals in purified SMNC-Exos, D-Exos, D-Exos/miR21i and D-Exos/miR21i-L17E at different excitation/emission (Ex/Em) wavelength. The SMNC-Exos and chol-miR21i contained in each sample were labeled by FITC and Cy5, respectively. (E) UV-vis spectra of the SMNC-Exos, Dox, chol-miR21i-Cy5, L17E and D-Exos/miR21i-L17E. The inset shows the magnified UV-vis spectra from 600 to 700 nm. (F) Gel retention assay of D-Exos/miR21i prepared with D-Exos and varied concentrations of cholesterol-conjugated miR-21i or non-cholesterol-conjugated miR-21i. The Cy5-labeled miR-21i bands are displayed. (G) Schematic of the methods for confirming the localization of chol-miR21i on D-Exos/miR21i. Cy5-labeled miR-21 is complementary to chol-miR21i on D-Exos/miR21i. If miR-21i is completely display on the surface of exosomes, Cy5-miR21 will bind to D-Exos/miR21i, otherwise it will not. (H) Analysis for the binding of Cy5-miR21 to D-Exos/miR21i by gel electrophoresis. The Cy5-labeled miR-21 bands are displayed here. (I) Release profiles of Dox from D-Exos/miR21i-L17E in PBS (pH=7.4) and acetate buffer (pH=5.0) for 24 h. Data in (I) are presented as the mean ± standard deviation (SD) from three independent experiments (n=3).

We rationally engineered blood exosomes to integrate Dox, chol-miR21i, magnetic-targeting molecules and L17E peptides, allowing the construction of blood exosome-based multifunctional co-delivery nanosystems. To obtain exosomes, first of all, anchoring multiple superparamagnetic nanoparticles onto per exosome through the transferrin (Tf)-Tf receptor interaction, blood exosome-based superparamagnetic nanoparticle cluster (SMNC-Exos) were separated from serum of healthy mice, which simultaneously integrated magnetic-targeting functional molecules into exosomes. Nanoparticle tracking analysis (NTA) and dynamic light scattering (DLS) measurements revealed that SMNC-Exos were physically homogeneous, with a narrow size distribution centered at 93 nm (Figure 1A and Figure S1). The representative transmission electron microscope (TEM) images (Figure 1A) showed that the morphology of SMNC-Exos was a spherical vesicle surrounded by several black spots, which represented the successful construction of cluster structures. Western blot results confirmed the expression of exosome markers proteins (CD63, CD81, CD9) and Tf receptor, which indicated that the vesicles are RTC-derived exosomes (Figure 1C). Next, by making full use of the natural lipid bilayer of exosomes, we assembled doxorubicin (Dox) and miRNA21 inhibitor (miR-21i) into the SMNC-Exos. The Nile Red fluorescence intensity increased as the SMNC-Exos concentration increased, confirming the hydrophobic domains present in SMNC-Exos (Figure S2). Therefore, lipophilic Dox were passively loaded into exosomes during co-incubation with SMNC-Exos to form Dox-loaded SMNC-Exos (D-Exos). A single-stranded nucleic acid with a conjugated cholesterol moiety can be tethered to the exosomal lipid bilayer 41-43. We anchored single-stranded miR-21i with a 5'-tetra (ethylene glycol) (TEG) spacer and cholesterol modification (chol-miR21i) to the exosomal membrane via co-incubation, constructing a blood exosome-based Dox/miR-21i co-loaded nanosystem (D-Exos/miR21i). We then introduced the cationic L17E peptide onto exosomes through electrostatic interactions to form D-Exos/miR21i-L17E. NTA and DLS measurements showed that, compared with the SMNC-Exos, there was a slight increase in the hydrodynamic diameter of D-Exos/miR21i-L17E, with a peak value of 106 nm (Figure 1B and Figure S3). Combinatorial analysis of TEM images (Figure 1B) and western blot results (Figure 1C) showed that D-Exos/miR21i-L17E still exhibited blood exosome-based superparamagnetic nanoparticle cluster structure, indicating the effective integration of magnetic molecules in this nanosystem. These results also demonstrated that our integration strategy well preserved the exosomal nanoscale vesicle structure and surface function proteins. In addition, there was almost no change in the concentration of exosomes before and after engineering, which was approximately 2.21✕10^11^ particles or 127 μg exosomes isolated from 1 mL serum (Figure S4). Analysis by DLS indicated that the surface zeta potential of purified D-Exos/miR21i-L17E increased from -19.7±1.8 mV of the D-Exos/miR21i to -6.8±1.5 mV, suggesting the successful integration of cationic L17E peptides onto exosomes (Figure S5). When suspended in PBS buffer at 4°C and in serum at 37°C, the size of D-Exos/miR21i-L17E showed no obvious change over 7 days, demonstrating high storage stability and application stability (Figure S6).

To further determine the co-loading of Dox and miR-21i in exosomes, we constructed this co-loading nanosystem using Cy5-labeled chol-miR21i (chol-miR21i-Cy5). After removing the excess free molecules via magnetic separation, the D-Exos/miR21i-L17E was evaluated in the fluorescence imaging system. As shown in Figure 1D, where the SMNC-Exos was labeled by fluorescein isothiocyanate (FITC) and Dox was intrinsically fluorescent, the D-Exos/miR21i-L17E significantly exhibited three fluorescence signals, indicating the effective and concurrent presence of Dox and miR-21i in this blood exosomes-based nanosystems. The UV-vis spectra (Figure 1E) of D-Exos/miR21i-L17E showed two more absorption peaks with Dox around 485 nm and chol-miR21i-Cy5 around 650 nm compared to which of SMNC-Exos, further supporting the evidence for the integration of Dox and miR-21i into the SMNC-Exos. Overall, these data demonstrated that the construction of D-Exos/miR21i-L17E enabled the integration of Dox, miR-21i, magnetic molecules and L17E peptides on exosomes. Comparing with traditional protocols to engineering exosomes based on genetic manipulation of donor cells or physical stimulation 44, the engineering strategy we designed was able to preserve the integrity and crucial components of exosomes, and integrated required functional molecules and therapeutic agents, forming the blood exosome-based multifunctional co-delivery nanosystems.

We further quantified the loading capacity of SMNC-Exos to Dox and miR-21i. According to the absorbance of Dox at 485 nm (Figure S7A), quantitative analysis indicated that the Dox loading capacity was 10.02%. Analysis by gel electrophoresis assay showed all samples prepared with D-Exos and chol-miR21i-Cy5 exhibited a well-defined band near the gel core, whose brightness gradually increased as increasing chol-miR21i concentrations (Figure 1F), suggesting the effective combination of miR-21i with high molecular weight exosomes. Moreover, miR-21i without cholesterol conjugates did not associate with exosomes and shifted to positive electrode, indicating the binding of miR-21i to exosomes is dependent on its cholesterol moiety. Based on the fluorescence intensity of chol-miR21i-Cy5 (Figure S7B), we measured that approximately 1839 chol-miR21i molecules were loaded per exosome in purified D-Exos/miR21i-L17E. These results indicated that, profiting from the natural lipid bilayer structure, blood exosomes could co-load hydrophobic drugs and cholesterol-modified RNAs with high efficiency. We noted that the loading ability of exosomes to chol-miR21i was about flat with the average amount of RNAs electroporated into exosomes as previously reported 31. As we know, electroporation process may be accompanied with RNA aggregation, leading to the overestimation of the RNA amount actually loaded into exosomes 45. Here, the co-loading strategy we used avoided any physical stimulation process that may cause RNA aggregation. By means of the favorable magnetic response and dispersibility of SMNC-Exos 39, D-Exos/miR21i-L17E was able to be efficiently purified, permitting improved estimation of cargos loaded to the exosomes.

Next, to confirm the orientation of chol-miR21i on exosomes, we used Cy5-labeled miR-21 (Cy5-miR21) that was complementary to miR-21i on D-Exos/miR21i (Figure 1G and Figure S8). As shown in Figure 1H, gel electrophoresis showed that most Cy5-miR21 shifted to the positive electrode and did not bind to the miR-21i on D-Exos/miR21i. This result indicated that a majority of miR-21i entered into exosomes in whole or in part rather than displayed on the surface of exosomes, which is consistent with reports that cholesterol promoted single-stranded RNA entry into exosomes 41. Serum digestion assay evidenced the stability of chol-miR21i on D-Exos/miR21i-L17E, which remained about 76% after 8 h in simulated body fluid (Figure S9), indicating the protection of D-Exos/miR21i-L17E against serum digestion. Moreover, *in vitro* drug release profile demonstrated that D-Exos/miR21i-L17E showed only 29% Dox release at pH 7.4 (physiological environment) in 4 h, in contrast, exhibited two-phase release kinetics in pH 5.0 (endo/lysosomes): a relatively rapid release (73% in 4 h), followed by a slow prolonged release (Figure 1I). The accelerated release of Dox from D-Exos/miR21i-L17E at pH 5.0 is mainly due to the protonation of amino group of Dox at low pH, thereby weakening its binding capacity with the lipid bilayer of exosomes, and eventually accelerating the release of doxorubicin. This indicated that D-Exos/miR21i-L17E could avoid the drugs burst release during blood circulation, and then rapidly and massively release the drugs after entering the endosomes of tumor cells.

### RNA transfection efficiency of SMNC-Exos modified with L17E peptide

**Figure 2:**
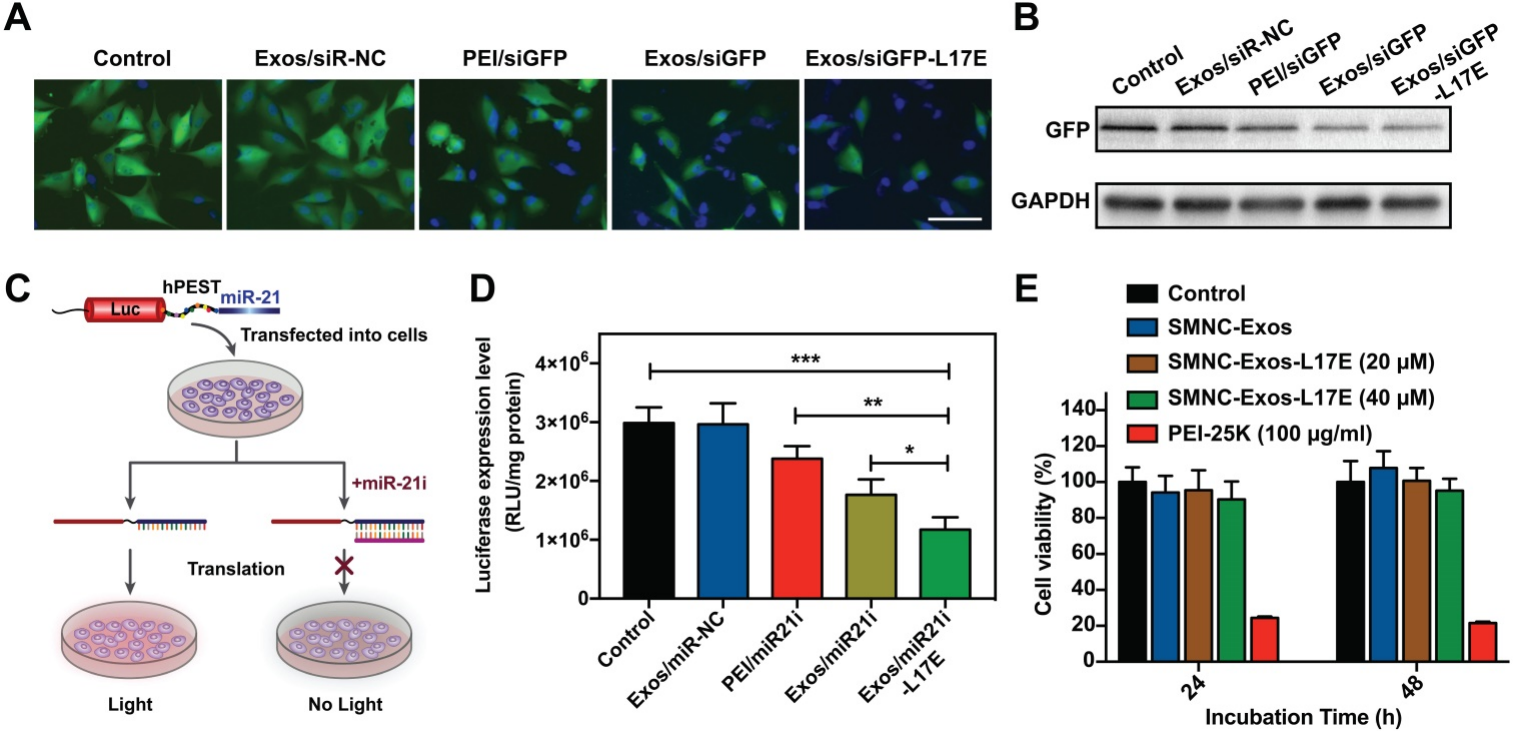
**RNA transfection ability of SMNC-Exos modified with L17E peptide.** (A) Fluorescence images of U87-GFP cells transfected with SMNC-Exos or SMNC-Exos-L17E carrying GFP siRNA (siGFP). The nucleus were stained with Hoechst 33342 (blue). The scale bar is 100 μm. (B) Western blot analysis of GFP protein expression. (C) Schematic representation of the luciferase reporter system (luc-hPEST-miR21). Luc: Firefly luciferase; hPEST: Protein degradation sequence (human-proline, glutamic acid, serine and threonine). Once exogenous miR-21i are transfected into cells via different nanocarriers and released into the cytoplasm, they block miR-21 associated with the 3'UTR of luciferase, which leads to decreased luciferase activity. (D) The bioluminescence of U87 cells after transfection of miR-21i into cells stably expressing luc-hPEST-miR21 by PEI, SMNC-Exos and SMNC-Exos-L17E. The results are expressed as RLU / mg protein. (E) Cell viability of U87 cells after treatment with SMNC-Exos containing different concentrations of L17E peptides for 24 h and 48 h. All data in (D) and (E) are presented as the mean ± SD from three independent experiments (n=3). The significant levels are shown as * p < 0.05, ** p < 0.01, *** p < 0.001.

Although exosomes enter cells through various pathway-mediated endocytosis 27, the problem of crossing the endosome membrane remains the same, resulting in a decrease in cargo delivery efficiency of exosome-based nanocarriers, especially for RNAs-containing delivery 46. To fully exploit its potential as drugs/RNAs co-delivery nanosystems, improving the RNA transfection efficiency of blood exosome is the key technological issue to solve. We investigated the gene transfection ability of SMNC-Exos modified with the L17E peptides. As a proof-of-concept, Exos/siGFP-L17E was constructed by electrostatically complexing siGFP-loaded SMNC-Exos (Exos/siGFP) with L17E, and then we evaluated its GFP fluorescence knockdown efficiency. As shown in Figure 2A, in the group treated with Exos/siGFP-L17E, the fluorescent images of the U87-GFP cells showed the most notable decrease in green fluorescence, while there was no significant reduction in the live cell number under this treatment (as shown by cell nucleus staining). The mean green fluorescence intensity significantly decreased in the cells treated with Exos/siGFP-L17E, down to 30.7% relative to untreated control, compared to the groups treated with PEI/siGFP (71.0%) and Exos/siGFP (48.1%) (Figure S10). Consistent with these results, the cells treated with Exos/siGFP-L17E showed the most effective downregulation of GFP expression (Figure 2B).

Next, we constructed a luciferase reporter system (luc-hPEST-miR-21), which contained a protein degradation sequence (human-proline, glutamic acid, serine and threonine, hPEST) 47 and triple repeated miR-21 sequence (Figure 2C). When miR-21i was transfected into U87 cells expressing this reporter gene with nanocarriers, we could evaluate the transfection efficiency by measuring the decrease degree of bio-luminescence signals. Therefore, as shown in Figure 2D, the greatest reduction in luciferase intensity occurred in the Exos/miR21i-L17E group, which was reduced by nearly 50% compared with that of the control. These results indicated that the modification of the L17E endosomolytic peptides on SMNC-Exos could effectively increase the gene transfection efficiency. As we know, some cationic polymers, such as polyethylenimine (PEI), has been widely designed as nucleic acids delivery carriers, because one of advantages is that its polycations can serve as 'proton sponges' to promote the release of oligonucleotides 48. However, the cytotoxicity and limited biodegradability of the polymer prevent its clinical use 49. As can be seen from our results, the SMNC-Exos exhibited a slightly better transfection efficiency than PEI, which benefited from the unique cell adhesion and endocytosis ability of exosomes as intercellular communicators. And after the modification of L17E peptide capable of disturbing the endosome membrane, the transfection efficiency was further improved. Moreover, the cell viability assay (Figure 2E) showed that SMNC-Exos containing different concentrations of L17E had no significant cytotoxicity, and the cells treated with such formulations exhibited higher cell viability (up to 90%) than PEI group. These results indicated that this modification strategy still retained the excellent biocompatibility of blood exosomes.

### Cellular uptake of D-Exos/miR21i-L17E

**Figure 3:**
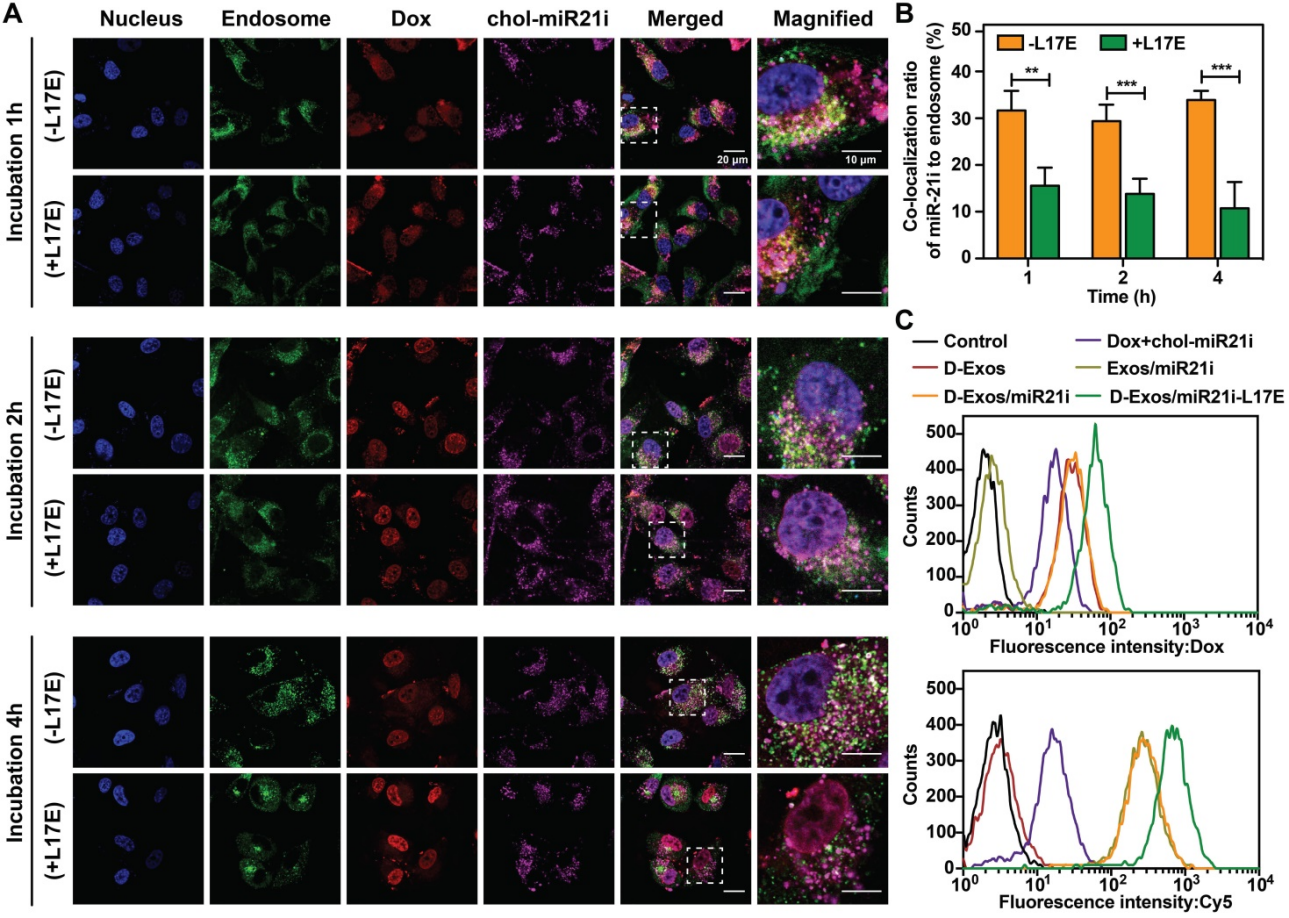
**Cellular uptake and intracellular distribution of D-Exos/miR21i-L17E.** (A) Confocal images of U87 cells incubated with D-Exos/miR21i and D-Exos/miR21i-L17E for different times (1 h, 2 h, 4 h). The fluorescence spots of Dox (red) and chol-miR21i-Cy5 (purple) were observed. The nucleus and endo/lysosome were stained with DAPI (blue) and Lysotracker Green (green), respectively. (B) Co-localization ratio of the miR-21i signals (purple) in D-Exos/miR21i and D-Exos/miR21i-L17E to endosome signals (green) at different time points. (C) Flow cytometry profile of U87 cells incubated with free Dox and chol-miR21i, D-Exos, Exos/miR21i, D-Exos/miR21i and D-Exos/miR21i-L17E for 4 h. The y-axis represents cell counts and the x-axis represents the Dox and chol-miR21i-Cy5 fluorescence intensity, respectively. Data in (B) are presented as the mean ± SD (n>50) from three independent experiments (n=3). The significant levels are shown as ** p < 0.01, *** p < 0.001.

To investigate whether the L17E peptides can enhance the endosome escape capabilities of blood exosome-encapsulated Dox and chol-miR21i, the intracellular trafficking of both pristine D-Exos/miR21i and D-Exos/miR21i-L17E was tracked in U87 cells using Lysotracker Green probe, which stains endo/lysosome. The intracellular distribution of the Dox (red) and miR-21i (purple) was tracked at different time points. As shown in Figure 3A, after incubation with the cells for 1 h, both Dox and miR-21i in the D-Exos/miR21i group showed efficient cellular uptake, but remained inside the endo/lysosomes, as indicated by the yellow dots (overlap of red and green dots) and white dots (overlap of purple and green dots) in the merged images. When analyzing the D-Exos/miR21i-L17E, the co-localization ratio of cargos to endosome was relatively low, suggesting that Dox and RNA began to escape from the endosome under L17E triggering. As the incubation time increased, the Dox in both groups gradually entered the nucleus, because small molecules could cross the biological membrane through a passive diffusion mechanism 50. As cellular uptake time increased, the internalized miR-21i molecules of D-Exos/miR21i treatment group gradually dispersed into cytosol, but were still partially entrapped in endosomes. Co-localization analysis of purple and green fluorescent spots showed that 34% of the RNA signal of D-Exos/miR21i co-localized with endosome when the incubation time reached to 4 h (Figure 3B). In contrast, at 4 h incubation with D-Exos/miR21i-L17E, significantly higher Dox fluorescence signal was observed in the cell nucleus. Simultaneously, the co-localization of miR-21i to endosome was significantly reduced, suggesting that the L17E modification enabled efficient endosomal escape of the Dox and miR-21i molecules. These results showed that the D-Exos/miR21i could deliver Dox and miR-21i into the nucleus and cytoplasm respectively, because the intracellular secretion and transport of exosomes enabling them to cross the endosomal membrane 25, but with undesirable efficiency. The introduction of L17E peptide could significantly promote the release of SMNC-Exos-encapsulated cargos from endosomes to cytosol, supporting the results that L17E modification improved the RNA transfection efficiency of SMNC-Exos.

Notably, in the group of D-Exos/miR21i-L17E, the cells showed stronger Dox and miR-21i uptake than D-Exos/miR21i. After incubation for 4 h, flow cytometry analysis was performed to further quantitate the cellular uptake of Dox and Cy5-miR21i. The corresponding fluorescence signals, including Dox and Cy5, and average fluorescence intensity (MFI) are presented in Figure 3C and Figure S11. The cells treated with D-Exos/miR21i-L17E exhibited the strongest Dox and Cy5 fluorescence signals. Compared with the D-Exos/miR21i group, the MFI of Dox and miR-21i increased approximately 1.8-fold and 2.0-fold, respectively, which may be due to the physiological effect of L17E that induces macropinocytosis 40. This eventually leads to an enhanced cellular uptake of the D-Exos/miR21i, modified with L17E, and then accelerates endosomal escaping, thereby increasing transfection efficiency.

### *In vitro* combination antitumor therapy

**Figure 4:**
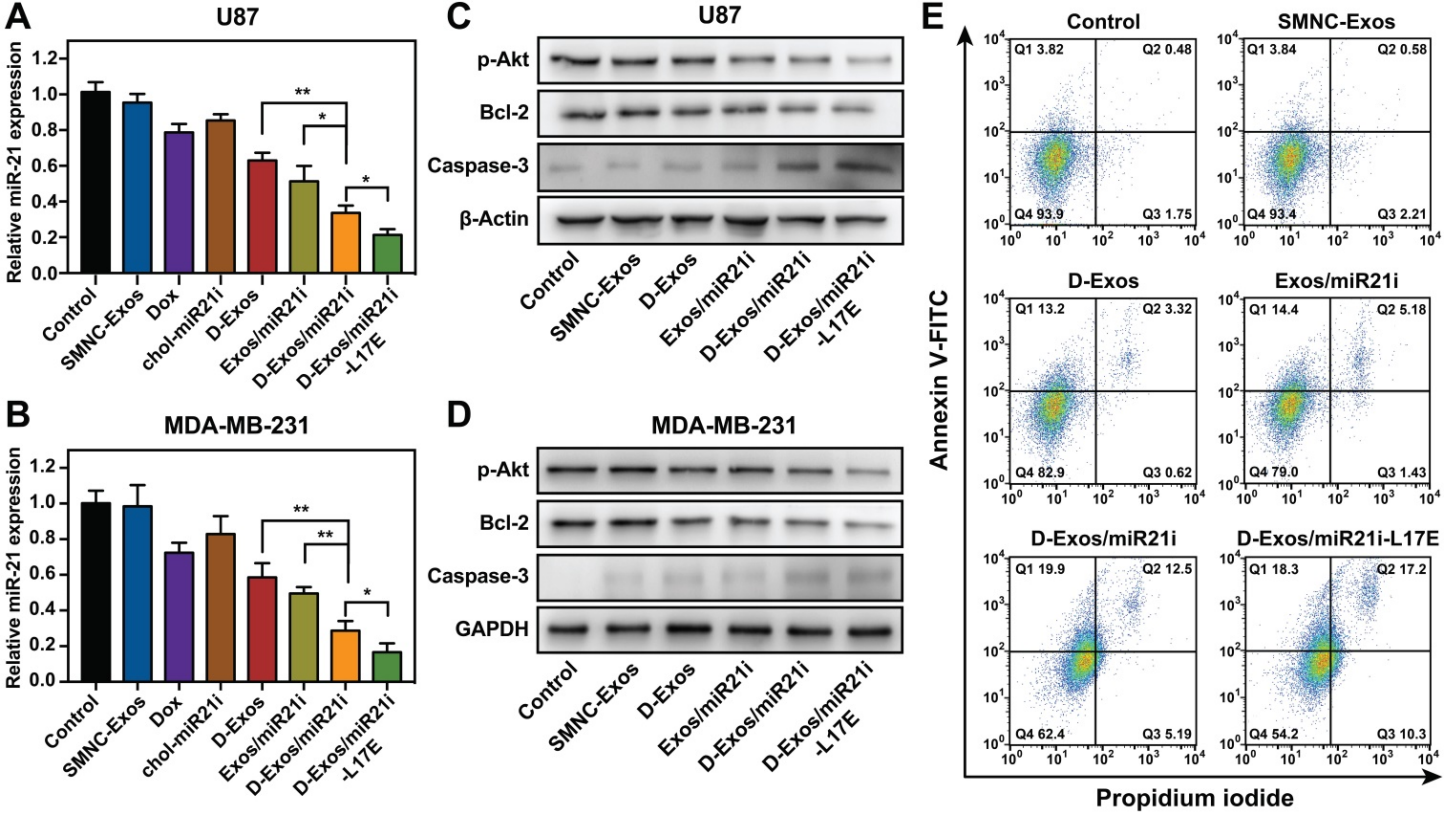
***In vitro* gene/chemo combination anticancer activity.** (A, B) Relative expression levels of miR-21 in U87 (A) and MDA-MB-231 (B) cells after treatment with different samples. (C, D) Western blot analysis of AKT, BCL-2 and Caspase-3 protein expression in U87 (C) and MDA-MB-231 (D) cells after incubating with PBS, SMNC-Exos, D-Exos, D-Exos/miR21i, and D-Exos/miR21-L17E, respectively. Cell cytoskeleton protein (β-Actin) and glyceraldehyde-3-phosphate dehydrogenase (GAPDH) are used as the loading control. (E) Cell apoptosis of U87 cells in each treatment group. Cell apoptosis ratios were assessed using flow cytometry. The data in (A, B) are presented as the mean ± SD from three independent experiments (n=3). The significance levels are shown as * p < 0.05, ** p < 0.01.

We then investigated the gene/chemo combination therapy effect of D-Exos/miR21i-L17E *in vitro*. Recent reports have identified that miR-21 is abnormally overexpressed in several types of cancer, and suppression of miR-21 can restrain the proliferation of cancer cells and render cancer cells more susceptible to chemotherapy drugs 51, 52. Downregulation of miR-21 expression was performed in U87 and MDA-MB-231 cancer cell lines. As shown in Figure 4A and B, compared to the untreated cells (blank control), cells treated with SMNC-Exos exhibited no obvious changes in the miR-21 expression. In Exos/miR21i group, the expression of miR-21 was significantly downregulated to 51.3% in U87 cells, confirming the feasibility of SMNC-Exos as RNA delivery vectors to down-regulate the expression of target genes. Moreover, the expression of miR-21 in cells treated with D-Exos/miR21i was lower than Exos/miR21i group, leaving only 33.7% in U87 and 28.6% in MDA-MB-231 cells, which is probably because Dox alone can reduce miR-21 transcription. Furthermore, a greater reduction of miR-21 expression was observed in the D-Exos/miR21i-L17E group, with only 21.3% remaining in U87 cells and 16.6% remaining in MDA-MD-231 cells. These results indicated that D-Exos/miR21i could effectively achieve the miR-21 downregulation, and more importantly, the modification of the L17E could maximize this effect.

According to the literature reports, miR-21 overexpression can promote the proliferation of cancer cells via increasing the expression of phosphorylated Akt and the anti-apoptotic proteins Bcl-2 53. For this reason, downregulation of endogenous miR-21 expression should decrease the expression of these proteins, and ultimately inhibit the proliferation of the cancer cells. The expression of p-Akt, Bcl-2 and Caspase-3 was assessed via Western blot analysis after cancer cells were treated with different samples for 48 h. As shown is Figure 4C and D, the D-Exos/miR21i groups showed significant increase of Caspase-3 and obvious decrease of p-Akt and Bcl-2, compared to the alone treated groups. More importantly, the greatest reduction of Bcl-2 and the largest increase of Caspase-3 were shown in the D-Exos/miR21i-L17E group, which was consistent with the downregulation level of miR-21. The quantitative assay demonstrated a 66% and 64% decrease in p-Akt and Bcl-2 level for D-Exos/miR21i-L17E, respectively, compared with the untreated cells, along with a 6.7-fold increase in Caspase-3 (Figure S12). These results again confirmed the enhanced delivery efficiency obtained from L17E-modified exosomes, thus expanding the Dox/miR-21i combination therapeutic effect. In addition, the protein expression levels of cells treated with SMNC-Exos had no significant differences compared to the control group, suggesting that blood exosomes itself had no therapeutic or cancer-stimulating activities.

To further assess the ability of D-Exos/miR21i-L17E to trigger tumor cell apoptosis, we analyzed U87 cells treated with different samples by flow cytometry. As expected, the cell apoptosis percentages of the D-Exos/miR21i group (37.6%) and D-Exos/miR21i-L17E group (45.8%) were significantly higher than that of other comparative groups, and there was also a significant difference between the two groups (Figure 4E and Figure S13). According to these results, we concluded that the engineered blood exosome-based co-delivery system could achieve significant tumor cell apoptosis, which attributed to the combination effects of the chemotherapeutic activity via Dox and the gene therapy activity via miR-21i, and the L17E further enhance the therapy effect.

### Tumor-targeting capability of D-Exos/miR21i-L17E

**Figure 5:**
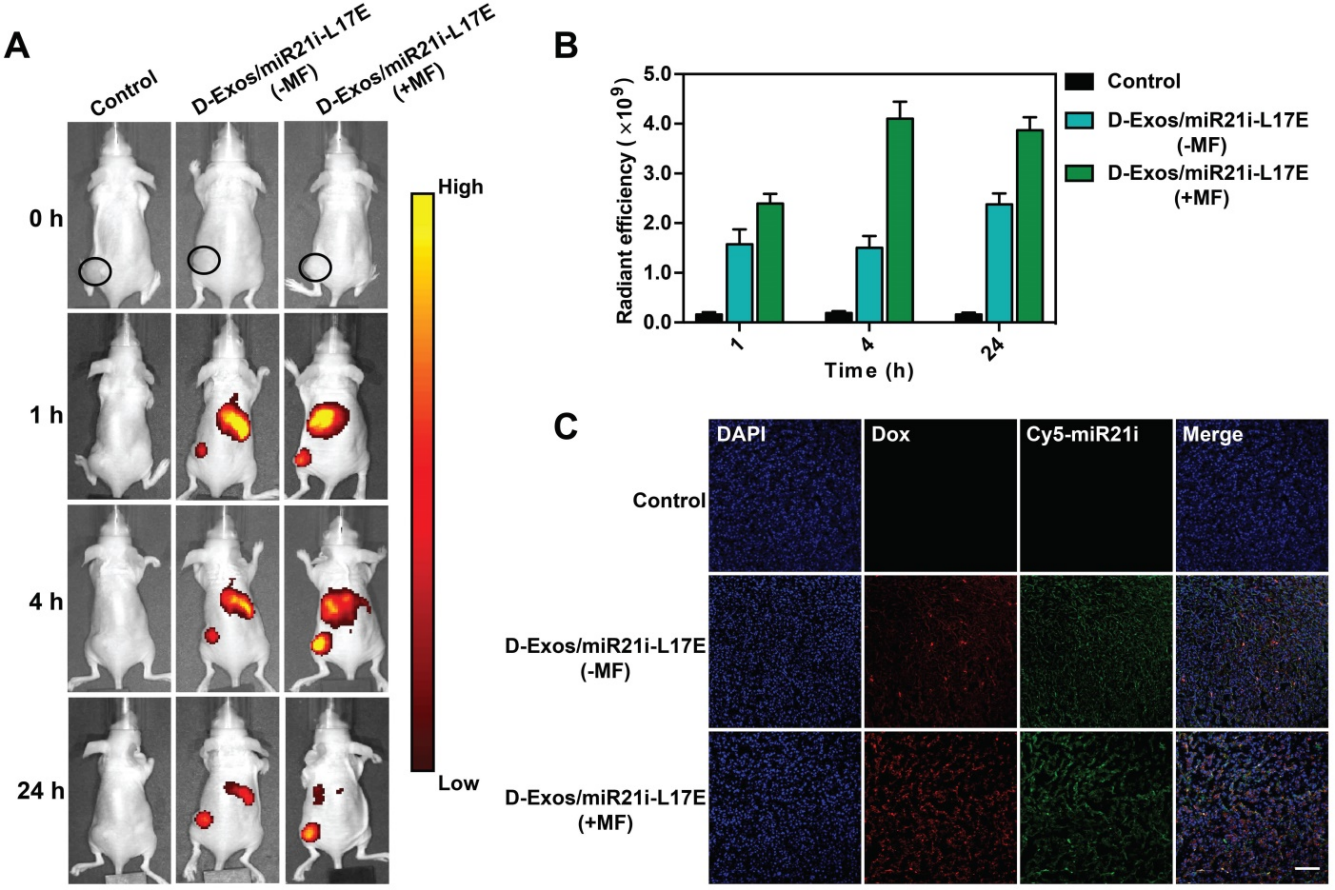
** Tumor targeting efficiency of engineered exosomes in tumor-bearing mice *in vivo*.** (A) *In vivo* NIRF imaging of U87 tumor-bearing nude mice after intravenous injection of Cy5.5-labeled D-Exos/miR21i-L17E with/without an external MF at 1 h, 4 h and 24 h post-injection. (B) Quantitative analysis of the tumor accumulation of the D-Exos/miR21i-L17E based on the fluorescence intensity from the *in vivo* images. (C) The accumulation of Dox and Cy5-miR21i in the tumor section, where red represents Dox, green represents miR21i and cell nucleus was stained by DAPI. The scale bar is 100 μm. Data in (B) are presented as the mean ± SD (n=5).

For chemo/gene combination antitumor therapy, it is essential for delivery systems to efficiently gather in tumor region after the administration. The blood circulation of the D-Exos/miR21i-L17E were investigated first, as shown in Figure S14, it was calculated to follow a two-compartment model blood circulation and the half-time was 7.61 h. The superb blood retention and circulation half-time made it more beneficial to tumor accumulation. To evaluate tumor accumulation efficiency, we labeled the D-Exos/miR21i-L17E with Cy5.5, for *in vivo* imaging after intravenous administration into tumor-bearing (U87) mice. At predetermined time points (1 h, 4 h and 24 h), the *in vivo* bio-distribution of D-Exos/miR21i-L17E was studied using an IVIS fluorescence imaging system. As shown in Figure 5A, the fluorescence signal was observed in the tumor site regardless of whether the magnetic field (MF) was applied. Considering the nanoscale size of D-Exos/miR21i-L17E, the effective tumor accumulation should be induced by the enhanced permeability and retention (EPR) effects 1, 24. Compared to the group without application of an MF, the D-Exos/miR21i-L17E (+MF) group exhibited the higher enrichment in the tumor. Quantitative analysis from the *in vivo* images indicated that strong fluorescence signals were detected at the tumor site with application of an MF (1T) only for 1 h. However, in the absence of an MF, the fluorescence intensity did not reach this level until 24 h after injection (Figure 5B). Moreover, at 4 h and 24 h after administration, the radiant efficiency of the engineered exosomes in tumors showed more significant differences between with or without MF application, which increased approximately 2.7-fold and 1.6-fold, respectively. Confocal laser scanning microscopy (CLSM) observation of the tumor sections (Figure 5C) indicated a substantially higher level of Dox and Cy5-labeled miR21i throughout the tumor tissue from the mice treated with D-Exos/miR21i-L17E (+MF) than that from the D-Exos/miR21i-L17E (-MF) group. The fluorescence signals of Dox (red) and miR21i (green) were mainly observed in the nuclei and cytoplasm, respectively, which was consistent with the results of the cell assay. These results indicated that D-Exos/miR21i-L17E had excellent tumor targeting ability under the application of MF, confirming the construction of SMNC-Exos did enhance the tumor accumulation of exosome, eventually increased the uptake of Dox and miR-21i by cancer cells.

### *In vivo* combination antitumor efficacy

**Figure 6:**
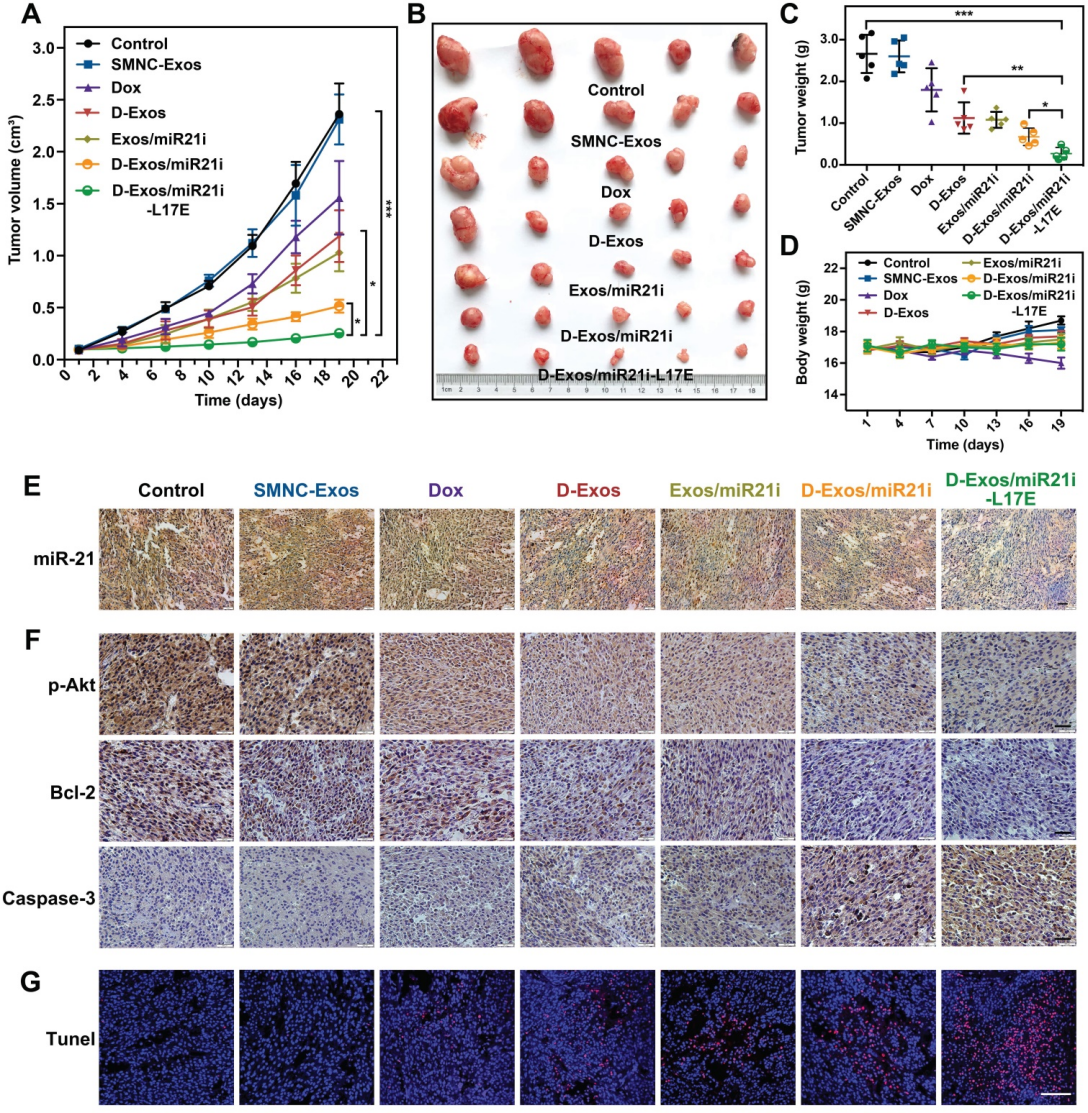
**Gene/chemo combination antitumor efficacy of D-Exos/miR21i-L17E *in vivo*.** (A) Tumor growth curves of U87 subcutaneous tumor in nude mice injected with PBS, SMNC-Exos, Dox, D-Exos, Exos/miR21i, D-Exos/miR21i and D-Exos/miR21i-L17E. Every mouse was applied with a MF of 1T. Tumor volume was examined every 3 days for 18 consecutive days. (B) *Ex vivo* photograph of the tumors from the treated mice at 18 days' post-injection. (C) Comparison of the weight of collected tumor tissues from the mice treated with different formulations. (D) Changes in body weight of the mice with different treatment. (E) RNA *in situ* hybridization presenting the expression of miR-21 in the tumor tissue from the mice of each treatment group. Nucleus is stained blue, and miR-21 is stained brown. The scale bar is 50 μm. (F) Immunohistochemistry analyses of the expression of p-Akt, Bcl-2 and Caspase-3 in each treatment group. Nuclei are stained blue, and the proteins are stained brown. The scale bar is 200 μm. (G) TUNEL analysis of the tumor tissues from the mice in each treatment group. Normal cell nuclei are stained blue, and apoptotic cell nuclei are stained red. The scale bar is 100 μm. Data in (A) are presented as the mean ± SEM, and data in (C, D) are presented as the mean ± SD (n=5). The significance levels are shown as * p < 0.05, ** p < 0.01, *** p < 0.001.

Based on the efficient enrichment of Dox and miR-21i at tumor sites, we investigated the potential of D-Exos/miR21i-L17E in gene/chemo combined antitumor therapy *in vivo*. Different drug formulations were injected into mice bearing U87 tumor via tail vein every three days for 18 days, and every mouse was applied with an MF. We continued to monitor the tumor volumes of mice from each group during the 18 days after treatment described above. As shown in Figure 6A, compared with the groups treated with PBS or SMNC-Exos, free Dox resulted in a slight inhibition of tumor growth, which was caused by the lower accumulation of Dox at the tumor site. The mice injected with the D-Exos/miR21i showed a more efficient antitumor effect than those with the D-Exos and Exos/miR21i treatment. More importantly, the most significant antitumor effect appeared in the D-Exos/miR21i-L17E group, which reduced the tumor volume by approximately 10 times at day 18 post-dosing compared with control groups. The excellent antitumor effects achieved here were attributed to the combined therapeutic effect of the Dox and miR21i on tumors and the enhanced tumor targeting ability of blood exosome-based superparamagnetic nanoparticle cluster. Meanwhile, the L17E peptides could promote the internalization of exosomes as well as facilitate the cytosolic release of Dox and miR-21i, leading to enhanced combined antitumor effects. This result was further confirmed by the *ex vivo* tumor morphology (Figure 6B) and the tumor weights of each group (Figure 6C). Moreover, no noticeable body weight loss was observed in the groups treated with different engineered blood exosome-based nanoformulations (Figure 6D), illustrating the satisfactory tumor-targeting and biocompatibility of the prepared co-delivery nanosystem.

Further studies of the tumor tissues with RNA *in situ* hybridization (FISH) and immunohistochemistry (IHC) assay confirmed that the tumor growth inhibition was associated with the Dox- and miR-21i-mediated gene transcription regulation. The FISH analyses showed that the tumor from the D-Exos/miR21i-L17E treatment group exhibited substantial decrease of miR-21 expression (Figure 6E), which was consistent with the *in vitro* experiments. At the molecular level, the expression levels of pAkt and Bcl-2 were significantly inhibited by the combined therapy of Dox and miR21i using D-Exos/miR21i-L17E, whereas the Caspase-3 showed the greatest increases compared with other groups (Figure 6F). By inhibiting the essential proteins of the PTEN-pAkt signaling pathways, D-Exos/miR21i-L17E effectively triggered the tumor cells apoptosis, which was confirmed by directly observing the tumor sections stained with terminal deoxynucleotidyl transferase dUTP nick end labeling (TUNEL) (Figure 6G). These results confirmed the successful down-regulation of oncogene expression and induction of tumor cells apoptosis *in vivo* achieved using engineered exosomes, suggesting the potential of D-Exos/miR21i-L17E as an effective strategy for gene/chemo combination therapy.

The histological changes of major organs and tumors induced by different nanoformulations were compared using the *ex vivo* hematoxylin and eosin (H&E) staining assay. As shown in Figure S15, compared to the control group, neither noticeable damage nor inflammation was observed in H&E staining images of tissues collected from SMNC-Exos treated group, indicating the negligible histological toxicity of SMNC-Exos itself. After treatment with D-Exos/miR21i-L17E, most of the tumor cells were destroyed and became apoptotic or necrotic, while there were lower degrees of damage in other groups, indicating, once again, the best therapeutic effect resulted from the combined therapy based on engineered exosomes we constructed. Meanwhile, the major organs of D-Exos/miR21i-L17E treatment group retained their normal morphology, indicating no noticeable side effects in the treated mice. We further evaluated the immunogenicity of *in vivo* use of engineered blood exosomes by measuring the inflammatory cytokine secretion from the mice treated with D-Exos/miR21i-L17E. No significant change in inflammatory cytokine secretion was observed (Figure S16), indicating the low immunogenicity of D-Exos/miR21i-L17E. These results demonstrated that exosomes-based multifunctional drugs/RNAs co-delivery nanosystems could strongly suppress tumor progression without any apparent adverse effects. In general, the engineered blood exosomes we constructed are eminently available co-delivery nanocarriers that can provide powerful new therapeutic modalities for antitumor combination therapy, and are promising tools for clinical treatment.

For clinical application, there is a continuing need to solve practical problems related to the safe manufacturing and quality control of such engineered blood exosomes. Considering the substantial quantities of exosomes required to achieve therapeutic effect, a safe and scalable mass production protocol is required at each stage of the production process 24. The safe and routine blood transfusions demonstrate the clinical translation potential of allogeneic blood exosomes for drug delivery 55. However, we still need to explore whether the administration of such allogeneic exosomes to cancer patients will have the potential immune response. Moreover, it is expected to minimize the invasiveness of this treatment strategy by exploring new manufacturing strategies to culture reticulocytes *ex vivo* to obtain abundant and biosafe exosomes. In the near future, the use of powerful yet facile technologies may enable us to better optimize and expand this engineered exosomes-based co-delivery nanosystem in a more controlled and precise manner, thereby advancing its clinical translation to drive the growth of exosomes-based personalized medicine.

## Conclusion

In summary, we reported a novel gene/chemo combination antitumor strategy that engineered blood exosomes as a nanoplatform capable of precise and potent co-delivery of hydrophobic drugs and nucleic acids to tumor cells. We demonstrated that the blood exosomes can flexibly and efficiently co-load hydrophobic drugs Dox and cholesterol-modified miR-21i by making full use of its natural lipid bilayer structure. *In vitro* studies elucidated that the introduction of L17E peptides could accelerate the endocytic uptake and endosomal escape of exosome-encapsulated cargos, thereby maximizing cargos delivery efficiency. Moreover, the nanoscale size and superparamagnetic nanoparticle clusters provided such co-delivery nanosystem with preferable tumor accumulation ability. With systemic administration of the D-Exos/miR21i-L17E, U87-bearing mice exhibited significantly enhanced tumor suppression and minor side effects. As far as we know, this is the first report of an application of the blood exosomes in cancer gene/chemo combination therapy. Considering the diversity of tumor suppressive pathways, this engineered exosome-based nanosystem can be further developed by replacing the therapeutic agent combination, demonstrating the potential of the blood exosome as a co-delivery nanocarrier, and it can broaden the therapeutic applications of exosomes further.

## Methods

### Materials

Carboxyl-group-functionalized superparamagnetic Fe_3_O_4_ nanoparticles were purchased from Nanjing XFNANO Materials Tech Co., Ltd. Dox, transferrin, nile red and FITC were obtained from Sigma-Aldrich (Shanghai, China). DAPI, Hoechst 33342 and Lysotracker Green were obtained from Invitrogen (USA). Cy5 and Cy5.5 fluorescence dyes were purchased from US Everbright^®^Inc (Suzhou, China). The 5'-cholesterol-TEG miR21i (sequences: 5'-UCAACAUCAGUCUGAUAAGCUA-3'), chol-miR21i-Cy5, Cy5-miR21 (sequences: 5'-UAGCUUAUCAGACUGAUGUUGA-3') and 5'-cholesterol-TEG siGFP (sequences: 5'-GGCUACGUCCAGGAGCGCACC-3') and were synthesized by Gene Pharma (Shanghai, China). L17E peptides (sequences: IWLTALKFLGKHAAKHEAKQQLSKL-amide) were synthesized by Nanjing GenScript Biotech Co., Ltd. The luc-hPEST-miR-21 reporter gene was synthesized according to the protocols with some modification (Shanghai Genechem Co., Ltd, China). The human glioma cell lines U87 and human breast cancer cell lines MDA-MB-231 were obtained from the China Academia Sinica cell repository (Shanghai, China). BCA protein assay kit was obtained from Solarbio Science & Technology (Beijing, China). Cell Counting Kit-8 (CCK-8) and Annexin V-FITC/PI apoptosis kit was purchased from Dojindo Chemical Technology Co. Ltd (Shanghai, China). Fluorescent TUNEL staining kit was obtained from Roche (Germany). All ELISA kits was purchase from Elabscience Biotechnology Co., Ltd (Wuhan, China). All reagents and solvents were purchased from Sigma-Aldrich and used as received unless otherwise noted.

### Preparation and characterization of D-Exos/miR21i-L17E

To construct SMNC-Exos, following the procedure we reported previously 39, superparamagnetic nanoparticle-Tf conjugation (M-Tf) were first synthesized by conjugation of holo-Tf to carboxyl-group-functionalized superparamagnetic Fe_3_O_4_ nanoparticle. Then, M-Tf solution (200 μL) was mixed pre-dialyzed fresh serum (1 mL) and incubated at 4 °C for 4 h, and SMNC-Exos were obtained from the mixture by magnetic separation using an MF (1T). Subsequently, for drugs loading, 20 μL of Dox hydrochloride (2 mg/mL) was added into the SMNC-Exos solution (100 μL, 1 mg/mL) with moderate stirring. After 30 min, 5 μL of triethylamine was added, and the solution was stirred for further 1h. Dox-loaded exosomes (D-Exos) was purified via magnetic separation. Then, D-Exos was gently shaken (500 rpm) with indicated concentrations of cholesterol-conjugated miR-21 inhibitor (chol-miR21i) for 1 h at room temperature in RNase-free PBS buffer. Unloaded chol-miR21i was removed by magnetic separation to obtain Dox/miR-21i co-loaded exosomes (D-Exos/miR21i). For preparation of final engineered exosomes (D-Exos/miR21i-L17E), endosomolytic peptide L17E was dissolved in DEPC water, and then the solution (40 μM) was mixed with D-Exos/miR21i at 37 °C for one hour.

For different samples, the size distribution and particle concentration were determined by recording and analyzing the Brownian motion of particles using a NanoSight NS300 system and Nanoparticles Tracking Analysis (NTA) software (Malvern, UK). For estimating the protein concentration of exosomes, a BCA protein determination assay was performed as described by the manufacturer. The high-resolution transmission electron microscope (JEM-2100F, JEOL Ltd., Japan) was used to observe the morphology and size of SMNC-Exos and D-Exos/miR21i-L17E, and the zeta potential of these nanoparticles was measured by Dynamic Light Scattering (DLS) (BI-90Plus, Brookhaven Instruments Ltd., USA).

The exosomal markers were confirmed by western blot analysis. In brief, SMNC-Exos, D-Exos/miR21i and D-Exo/miR21i-L17E were lysed with radio-immunoprecipitation assay (RIPA) buffer (Solarbio, Beijing, China). The sample proteins were separated via the sodium dodecyl sulfate polyacrylamide gel electrophoresis (SDS-PAGE) and transferred to polyvinylidene difluoride (PVDF) membranes. Membranes were blocked with 5% bovine serum albumin (BSA) at room temperature for 1 h, then incubated with CD9, CD81, CD63 and Tf receptor antibodies (1:1000 dilution; Cell Signaling Technology, Shanghai, China) in PBS-Tween 20 (PBST) buffer at 4 °C overnight. Subsequently, the membranes were washed in PBST for three times and incubated with a horseradish peroxidase (HRP) - linked secondary antibody (1:10000; Promega, USA) at room temperature for 1 h followed by PBST washing. The expected bands were visualized on a Synege G: BOX Chemi XT4 chemiluminescent imaging system.

### Stability of D-Exos/miR21i-L17E

Purified D-Exos/miR21i-L17E were transferred into glass vials to incubate in PBS at 4°C buffer and serum at 37°C. At selected time intervals, D-Exos/miR21i-L17E were separated magnetically and were re-dispersed in PBS buffer. The particle size of two samples was evaluated using DLS. Measurements of the two groups were taken in triplicate, and results were averaged.

### Optical measurements

All samples were purified by magnetic separation. SMNC-Exos, D-Exos, D-Exos/miR21i and D-Exos/miR21i-L17E solutions were dropped in transparent 96-well plates respectively and put in the fluorescence imaging chamber. The fluorescence signals were collected using the IVIS imaging system (Caliper Life Sciences, USA) with different Ex/Em wavelengths. Here, the SMNC-Exos and chol-miR21i contained in each sample were labeled with FITC and Cy5, respectively. In addition, for each material and D-Exos/miR21i-L17E, the UV-vis spectra were collected with a UV-vis spectrophotometer (UV-1800, Shimadzu, Japan) under a range of 200-800 nm.

### Quantitation of Dox and miR-21i loaded into exosome

When 40 μg Dox was added into 100 μg of exosomes and D-Exos/miR21i-L17E were obtained *via* magnetic separation, the amount of Dox loaded into engineered exosomes was measured and calculated from a calibration curve based on the absorbance intensity at 485 nm using a UV-Vis spectrophotometer. Then, the chol-miR21i-Cy5 loading capacity was assessed by agarose gel electrophoresis, which was carried out on 1% agarose gel with a constant voltage of 100 V for 15 min in TAE buffer. The images were acquired by a gel imaging system (Protein Simple, USA). When 5 μM chol-miR21i-Cy5 was incubated with 50 μg exosomes, quantitation of chol-miR21i-Cy5 loaded into exosomes was performed by measuring the fluorescence using a TECAN spectrophotometer (Infinite M200 Pro, Männedorf, Switzerland) and calculating from a concentration standard curve. The number of chol-miR21i loaded per exosome was estimated by dividing the total amount of retained chol-miR21i by the number of exosomes detected by NTA.

### Localization of chol-miR21i on exosomes

The Cy5-miR21, which was equivalent and complementary to chol-miR21i loaded on exosomes, was incubated with 100 μg of D-Exos and D-Exos/miR21i in 200 μL RNase-free PBS buffer, respectively. The Cy5-miR21 tethered onto D-Exos/miR21i was analyzed using 1% agarose gel electrophoresis, thus determining whether chol-miR21i entered the exosomal lumen.

### FBS digestion experiments

The purified D-Exos/miR21i-L17E (1 mL) with chol-miR21i-Cy5 were mixed with 100 μL of FBS (Sigma) and incubated at 37 °C for 8 h. At certain time intervals, the samples were removed for fluorescence spectroscopy analysis. The miR-21i retention ratio was determined according to the fluorescence of Cy5-chol-miR21i.

### Drug release assay

With PBS (pH 7.4) and acetate buffer (pH 5.0), the release of Dox from D-Exos/miR21i-L17E was demonstrated using dynamic dialysis methods. Loaded in the dialysis tube (cutoff = 14 kDa), the sample (4 mL) was then immersed in 10 mL of buffer at 37 °C in a water box at a constant temperature. At certain time intervals, the dialysate was removed and replaced with a fresh buffer, and then analyzed by UV-vis spectrophotometer. Dox concentrations were determined according to standard curves at the corresponding buffer solutions.

### GFP silencing study

The Exos/siRNA and Exos/siRNA-L17E complex (L17E final concentration, 40 μM), prepared using GFP-targeted siRNA (siGFP), were incubated with GFP-U87 cells in 12-well plates (siRNA concentration, 50 nM). PEI at 25 kDa and non-targeting siRNA (siR-NC) were used as a positive and negative control, respectively. After incubation for 48 h, cell nucleus were stained with Hoechst 33342, a living cell nucleus-specific dye. The cell images were obtained using Confocal laser scanning microscopy (Olympus FluoView FV1200, Tokyo, Japan), and the fluorescence intensity was measured by ImageJ. Five representative cell images (>50 cells) were used to calculate the fluorescence intensity. Next, the expression of GFP protein was measured by Western blot analysis as described above but with anti-GFP antibody.

### *In vitro* luciferase reporter assay

U87 cells which stably expressed the luciferase reporter genes were seeded in 12-well plates. Then, different nanoformulations containing miR-21i (final concentration, 200 nM) were added to each well, and the cells were incubated for further 8 h. Luciferase activity was determined using a Bright-Glo TM Luciferase Assay System (Promega). The bioluminescence intensity was measured using a microplate reader (Synergy2, Bio-Tek, USA) and quantified as RLU/mg protein.

### Cell viability assay

The cytotoxicity of L17E peptide-modified SMNC-Exos was evaluated by a CCK-8 viability assay, using 25 kDa PEI as a control. Briefly, cells were seeded into 96-well plates at a density of 5×10^3^ cells/well and incubated overnight at 37 °C in 100 μL DMEM (Gibco) with 10% FBS v/v (Gibco). Subsequently, the culture medium in each well was replaced with fresh medium that contained Exos-L17E (L17E final concentration, 20 μM and 40 μM). After culture for an additional 24 and 48 h, the cells were rinsed using PBS buffer, followed by the addition of 100 μL CCK-8 working solution and another 2 h incubation. Quantification of the cell viability was done by measuring the absorbance at 450 nm with a TECAN microplate reader. The cell viability was calculated by referring to that of the cells without treatment.

### Cellular uptake study

U87 cells were seeded on the glass coverslips of a 12-well plate in 1 mL DMEM supplied with 10% FBS for 24 h. D-Exos/miR21i and D-Exos/miR21i-L17E contained chol-miR21i-Cy5 were subsequently introduced and incubated with cells for 1 h, 2 h and 4 h, respectively. To investigate the endosome escape of the Dox and miR-21i, the samples were gently washed twice with PBS to remove excess nanoparticles, and LysoTracker Green was added to the culture media (final concentration, 100 nM) and incubated for 1 h following the protocol for endo/lysosome staining. Afterwards, the cells were washed three times with PBS, fixed with paraformaldehyde for 15 min, and counterstained with DAPI (10 μg/mL) for 10 min to stain the nucleus. The intracellular distributions of Dox and chol-miR21i were observed and imaged by CLSM (Olympus FV1200). Colocalization ratios of miR-21i to endo/lysosome were obtained from ImageJ software. Five representative cell images (>50 cells) were used to calculate the colocalization ratios. The cellular uptake efficiency was also assessed using flow cytometry analysis. Briefly, different samples with the equal amount of Dox and chol-miR21i-Cy5 were added to the U87 cells and incubated for 4 h at 37 °C. After washed three times with PBS, the cells were collected after treatment with trypsin, followed by centrifugation (1000 rpm, 5 min). The cells were finally fixed with 4% paraformaldehyde, and their fluorescence was measured by flow cytometry (FACSVerse, BD, USA). All of these experiments were repeated in triplicates.

### Real-time PCR

RT-PCR was performed to study the *in vitro* downregulation of miR-21 expression with different treatments in cancer cells. Briefly, U87 and MDA-MB-231 cells were seeded into 6-well plates at a density of 2✕10^5^ cells per well and incubated overnight in 2 mL DMEM with 10% FBS v/v. Then, the culture medium was replaced with the fresh medium containing different nanoformulations. After 48 h incubation, total RNA was harvested from cells using TRIzol Reagent (Invitrogen, USA). A stem-loop-specific primer (GenePharma, Shanghai, China) was used to measure the expression levels of miR-21. The miRNA was converted to cDNA using the PrimeScript RT reagent kit (TaKaRa, Tokyo, Japan). cDNAs were quantified by SYBR Green (Thermo, USA) in a Real-Time Fluorescent Measurement System (QuantStudio 3, Thermo). Fold changes for the expression levels of miR-21 were calculated using the comparative cycle threshold (CT) method (2^-ΔΔCT^). Amplification of U6 was used as an internal control.

### Western blot assay

The U87 and MDA-MB-231 cells were seeded into 6-well plates for 24 h to bring the cells to the desired confluence. Cells treated with PBS, SMNC-Exos, D-Exos, Exos/miR21i, D-Exos/miR21i, and D-Exos/miR21i-L17E (with the Dox concentration 0.98 μg/mL and/or miR-21i concentration 52 nM) for another 48 h. After the transfection, each group of cells was washed with PBS and then lysed in cell lysis buffer supplemented with PMSF for 20 min at 4 °C. Then the homogenates were collected and protein concentrations were determined with a BCA assay kit. Total protein lysates were separated by SDS-PAGE on 10% SDS acrylamide gels, which was then transferred to PVDF membrane. The membranes were incubated with primary antibodies against p-Akt, Bcl-2 and Caspase-3 (1:1000 dilution; Abcam, UK) overnight, followed by incubating with an HRP-conjugated secondary antibody for 1 h. β-Actin and GAPDH (1:1000 dilution; Cell Signaling Technology, Shanghai, China) was set as a loading control. The density of target protein signals was visualized by a gel imaging system (Syngene G: BOX Chemi XT4).

### Evaluation of cell apoptosis

Tumor cell apoptosis was evaluated by flow cytometry analysis. The U87 cells were seeded in 6-well plates at a density of 2✕10^5^ cells per well. After treatment with different groups as shown above for 48 h, cells were collected and re-suspended in 100 μL ice-cold binding buffer, followed by the addition of 5 μL of Annexin V-FITC and 5 μL of PI solution. The samples were incubated for 15 min in the dark at room temperature, and then, fluorescence was measured by flow cytometry using a FACS flow cytometer (BD, USA). The cells treated with PBS under the same processes were used as controls.

### Blood circulation and tumor targeting study *in vivo*

Four- to six-week-old female BALB/c nude mice were obtained from the Animal Center of the Cancer Institute of Chinese Academy of Medical Science. All experimental protocols were conducted within the Tianjin Medical University Animal Research Guidelines and were approved by the Institutional Animal Care and Use Committee. The tumor-bearing mice were generated by subcutaneous injection of U87 cells (5 × 10^6^ for each mouse) on the dorsal side of the mice. For blood circulation analysis, U87 tumor-bearing mice were intravenous injected with Cy5-labeled D-Exos/miR21i-L17E (5 mg/mL, 200 μL). Orbital venous blood (50 μL) from each mouse was collected at different time points injection (0.5, 1, 2, 4, 8, 12, 24, and 48 h). The fluorescent intensity of the samples was tested by a fluorescence spectrometer. For tumor targeting study, when the tumor volumes reached to ~500 mm^3^, the mice were randomly divided into three groups. One group was tail intravenous injected with Cy5.5-labeled D-Exos/miR21i-L17E solution (5 mg/mL, 200 μL per mouse), and then, a magnet (MF density: 1 T) was placed over the surface of the tumor using Steri-Strip tape. Another group was injected with equal Cy5.5-labeled D-Exos/miR21i-L17E without a magnet, whereas the control group was injected with PBS. At 1 h, 4 h and 24 h post-injection, the mice were imaged using an IVIS Spectrum imaging system (Caliper Life Sciences, USA). The results were analyzed using Living Image 3.1 software. For determine the distribution of Dox and miR-21i in tumor, the tumor-bearing mice were treated as mentioned above, but Cy5-labeled miR21i was used here. After 24 h, the tumor tissues were isolated and embedded in optimal cutting temperature compound (OCT), followed by rapid freezing in -80 °C for 24 h. The frozen sections were dissected into 7 μm histology slices and stained with 4′,6-diamidino-2-phenylindole (DAPI). Images were captured using CLSM (Olympus, FV1000).

### *In vivo* antitumor efficacy

The tumor-bearing mice were established as described above. When the tumor volume was about 100 mm^3^ at 10 days after cell implantation, the mice were randomly divided into seven groups (n=5) and a magnet with a magnetic field of 1T was attached to them. The groups were PBS, SMNC-Exos, Dox, D-Exos, Exos/miR21i, D-Exos/miR21i and D-Exos/miR21i-L17E. Each tumor-bearing mouse was intravenously injected with various samples (5 mg/mL, 200 μL) every three days for 18 days (5 mg Dox equivalent and/or 5 nmol miR-21i per kg body weight for each dose). The changes in the tumor volume and body weight of mice were recorded. The estimated volume was calculated according to the formula: tumor volume (cm^3^) = 0.5 × length × width^2^. The largest longitudinal-section and cross-section of each tumor was taken to ensure the same condition. After finishing the treatment, the mice were euthanized, and major organs were harvested. We photographed the tumors and measured their average masses.

### Fluorescence *in situ* hybridization (FISH) detection of miR-21 of tumor tissues

FISH assay was performed on freshly frozen tumor tissue sections. In brief, slices were washed with PBS containing 0.5% Triton X-100. The slices were then incubated with the hybridization solution, which containing 1% blocking solution and anti-miR-21 oligonucleotide probes, in humid chamber at 37 °C overnight. After the incubation, the slices were washed each at 42 °C with 0.1% Tween-20 in 4×, 2× and 1× sodium citrate buffer (SSC) in dark. After rinsing with PBS for three times at room temperature and staining with hematoxylin, the slices were observed using a microscope (CX41, Olympus) for evaluating the expression level of miR-21.

### Immunohistochemistry, immunofluorescence and H&E analysis

All tissues were stored overnight in 4.0% (v/v) paraformaldehyde solution, followed by incubating with different concentrations of alcohol to remove moisture. The tissues were embedded in paraffin, and then dissected into 7 μm tissue slices. Immunohistochemistry (IHC) was performed to analyze the expression levels of p-Akt, Bcl-2 and Caspase-3. Histology slices were incubated with primary antibodies (1:100 dilutions) overnight at 4 °C, following by a biotin-labeled secondary antibody (1:100 dilutions) for 1 h at 37 °C, and then incubated with ABC-peroxidase and diaminobenzidine (DAB) and counterstained with hematoxylin. For analysis of tumor cell apoptosis, the tumor slices were stained with 50 μL TUNEL reaction mixture (Roche, Germany) for 60 min at 37 °C according to the manufacturer's protocol. The cell nuclei were visualized by staining with DAPI. All tissue sections, including heart, liver, spleen, lung, kidney, and tumor, were stained with Hematoxtlin and Eosin (H&E) to analyze the tumor and tissue pathological changes. All the images were captured using an inverted fluorescence microscope (CX41, Olympus).

### Immunogenicity evaluations assessed with inflammatory cytokine secretion

Ten KunMing mice of 8-10-week-old were divided into 2 groups and injected with 100 μL of PBS and engineered exosomes (1 mg) per mouse via tail vein every three days for 10 days. The blood samples were collected from the mice using coagulant tube, which allows the blood to coagulate naturally, and then centrifuged at 2000 rpm for 10 mins. The serum was collected and the levels of IFN-γ, IL-6, TNF-α was analyzed by enzyme linked immunosorbent assay (ELISA) following the protocol as provided in our previous research 54.

### Statistical Analysis

Statistical analysis was performed by a one-way ANOVA followed by Dunnett's post-test using GraphPad Prism 7.0 software. Data sets were compared using t tests with a two-tailed p value. p < 0.05 was considered statistically significant.

## Supplementary Material

Supplementary figures and tables.
