# Supplementary material for: Engineering blood exosomes for tumor-targeting efficient gene/chemo combination therapy
Source: Theranostics. 2020 Jun 22;10(17):7889–905. doi: 10.7150/thno.45028 (PMC7359100; doi:10.7150/thno.45028)
Supplement: Supplementary file 1 — Supplementary figures and tables. [file thnov10p7889s1.pdf]

Supplementary Material

Engineering blood exosomes for tumor-targeting efficient gene/chemo combination therapy

Qi Zhan[1,#], Kaikai Yi[2,#], Hongzhao Qi[3], Sidi Li[1], Xueping Li[1], Qixue Wang[2], Yunfei Wang[2], Chaoyong Liu[2], Mingzhe Qiu[2], Xubo Yuan[1], Jin Zhao[1,*], Xin Hou[1,*], Chunsheng Kang[2,*]

1. Tianjin Key Laboratory of Composite and Functional Materials, School of Material Science and Engineering, Tianjin University, Tianjin 300072, China
2. Department of Neurosurgery, Tianjin Medical University General Hospital, Laboratory of Neuro-oncology, Tianjin Neurological Institute, Key Laboratory of Post-Neurotrauma Neuro-Repair and Regeneration in Central Nervous System, Ministry of Education and Tianjin City, Tianjin 300052, China
3. Institute for Translational Medicine, Qingdao University, Qingdao, 266021, China

[#] These authors contributed equally to this work.

* Corresponding authors: Jin Zhao, E-mail: zhaojin@tju.edu.cn; Xin Hou, E-mail: houxin@tju.edu.cn; Chunsheng Kang, E-mail: kang97061@tmu.edu.cn

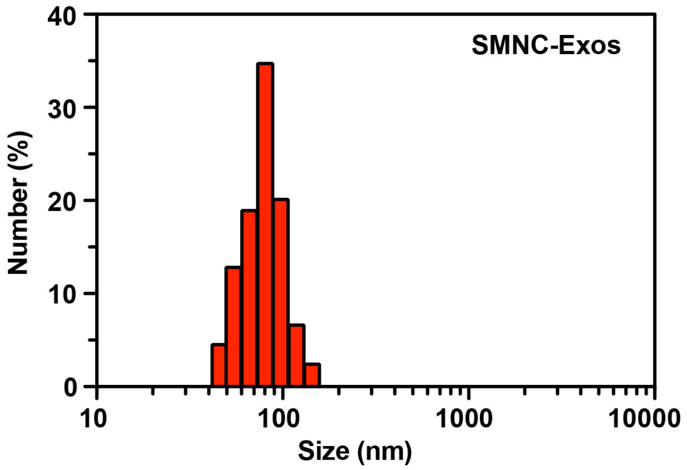

Figure S1. The dynamic light scattering (DLS) measurements of SMNC-Exos.

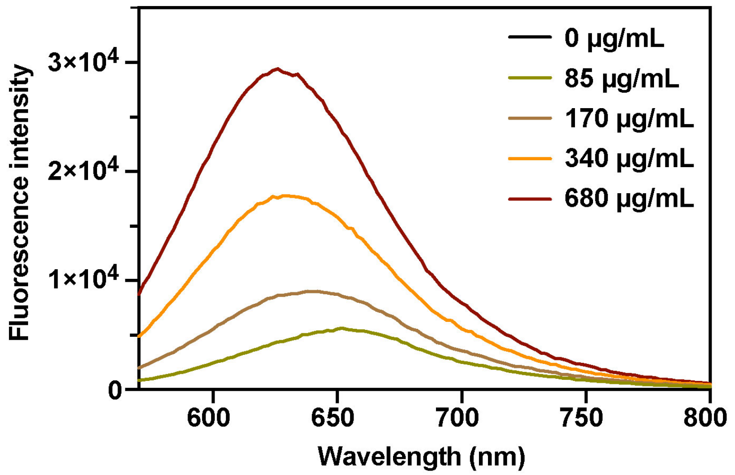

Figure S2. The fluorescence spectra of different concentrations of SMNC-Exos after incubation with Nile Red.

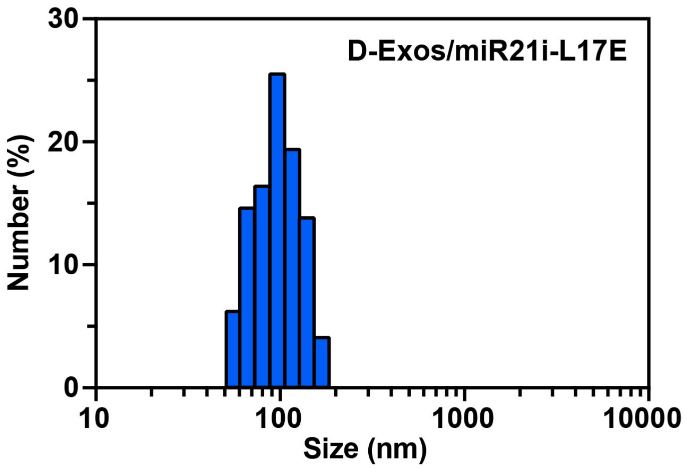

Figure S3. The dynamic light scattering (DLS) measurements of D-Exos/miR21i-L17E.

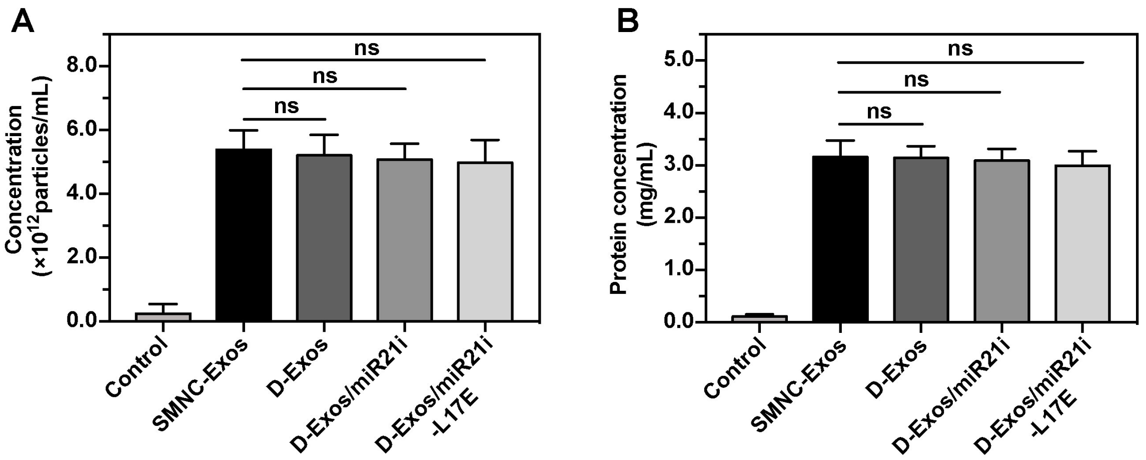

Figure S4. Comparison of particle concentration (A) and protein concentration (B) after each step of magnetic separation. Data are shown as the mean ± SD (n=3). The significance levels are shown as ^{ns} $p > 0.05$.

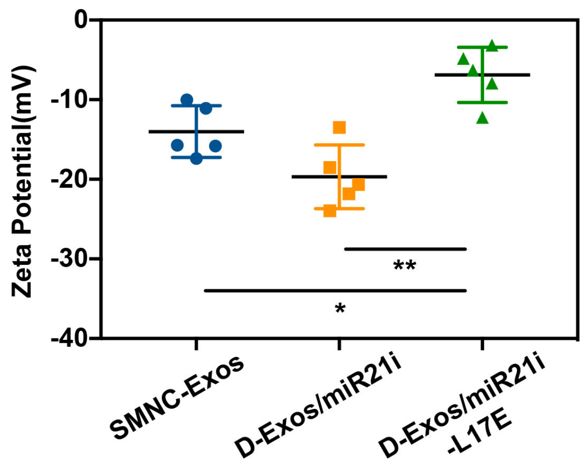

Figure S5. The zeta potential variation of SMNC-Exos, D-Exos/miR21i and D-Exos/miR21i-L17E. Data are shown as the mean ± SD (n=5). The significance levels are shown as * $p < 0.05$, ** $p < 0.01$.

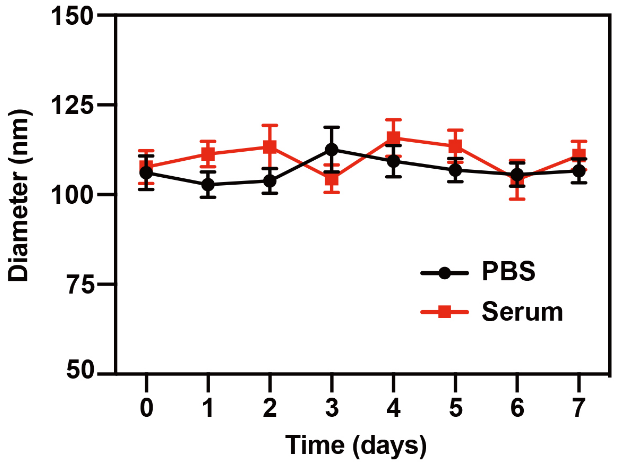

Figure S6. Stability of D-Exos/miR21i-L17E at 4°C in PBS buffer and at 37°C in serum, determined by monitoring particle size (diameter) over 7 days. Data are presented as the mean ± SD (n=3).

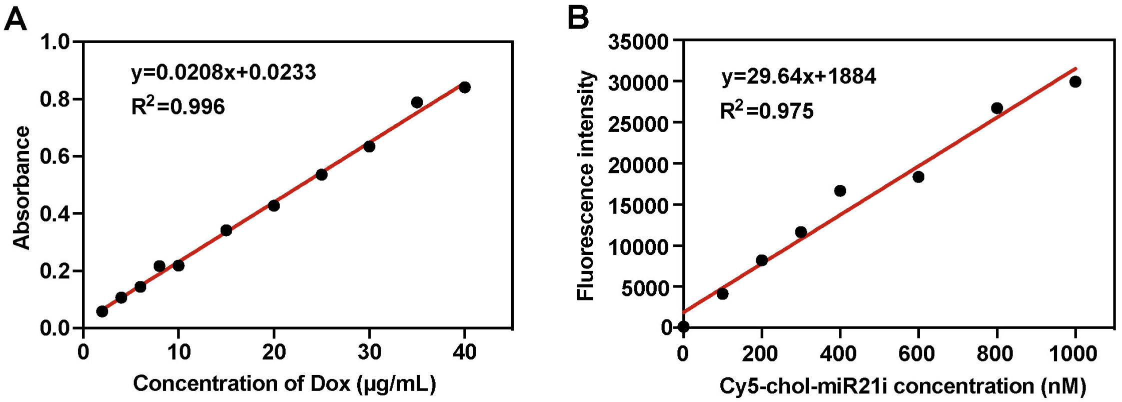

Figure S7. (A) Concentration standard curve of Dox, measured by UV-Vis spectrophotometer. (B) Concentration standard curve of Cy5-chol-miR21i, measured by fluorescent intensity.

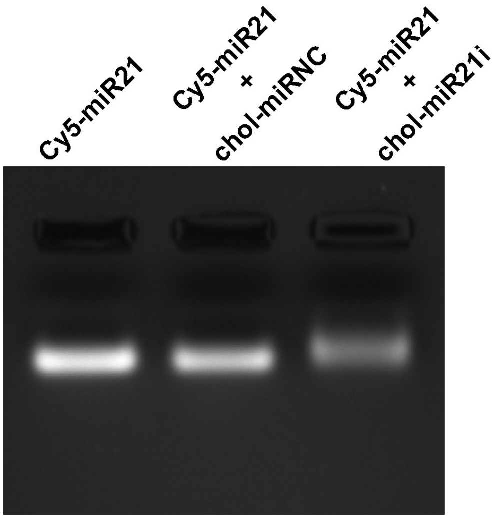

Figure S8. Gel electrophoresis analysis of the complementarity of Cy5-miR21 and chol-miR21i.

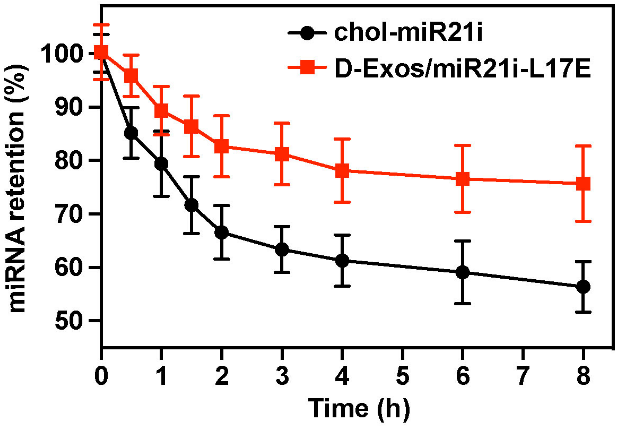

Figure S9. Percentage of chol-miR21i associated with the D-Exos/miR21i-L17E after incubation with 10% v/v FBS for 8 h at 37 ℃. The free chol-miR21i as the control. Data are presented as the mean ± SD (n=3).

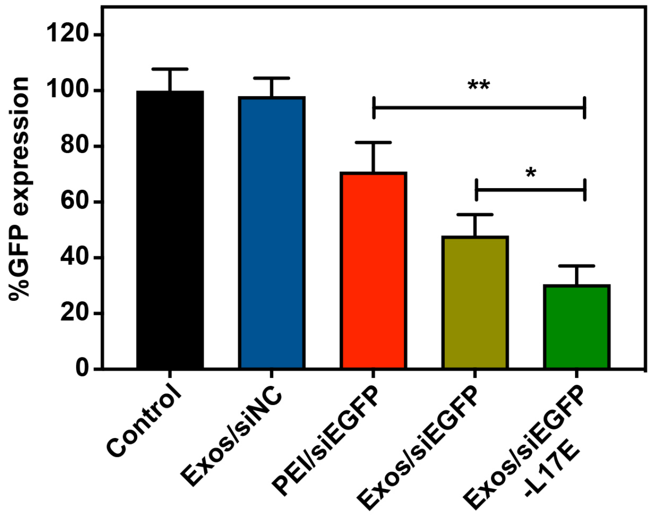

Figure S10. GFP knockdown efficiency was determined based on the fluorescence images of U87-GFP cells transfected with different samples using ImageJ software. Data are presented as the mean ± SD (n>50) from three independent experiments (n=3). The significance levels are shown as * $p < 0.05$, ** $p < 0.01$.

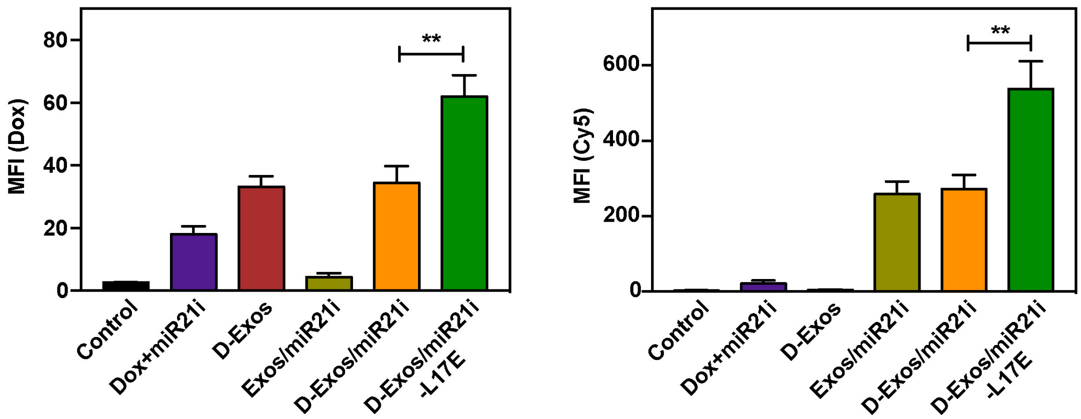

Figure S11. Quantification of cell internalization shown by the mean fluorescence intensity (MFI). Data are presented as the mean ± SD from three independent experiments. The significant levels are shown as * $p < 0.05$, ** $p < 0.01$.

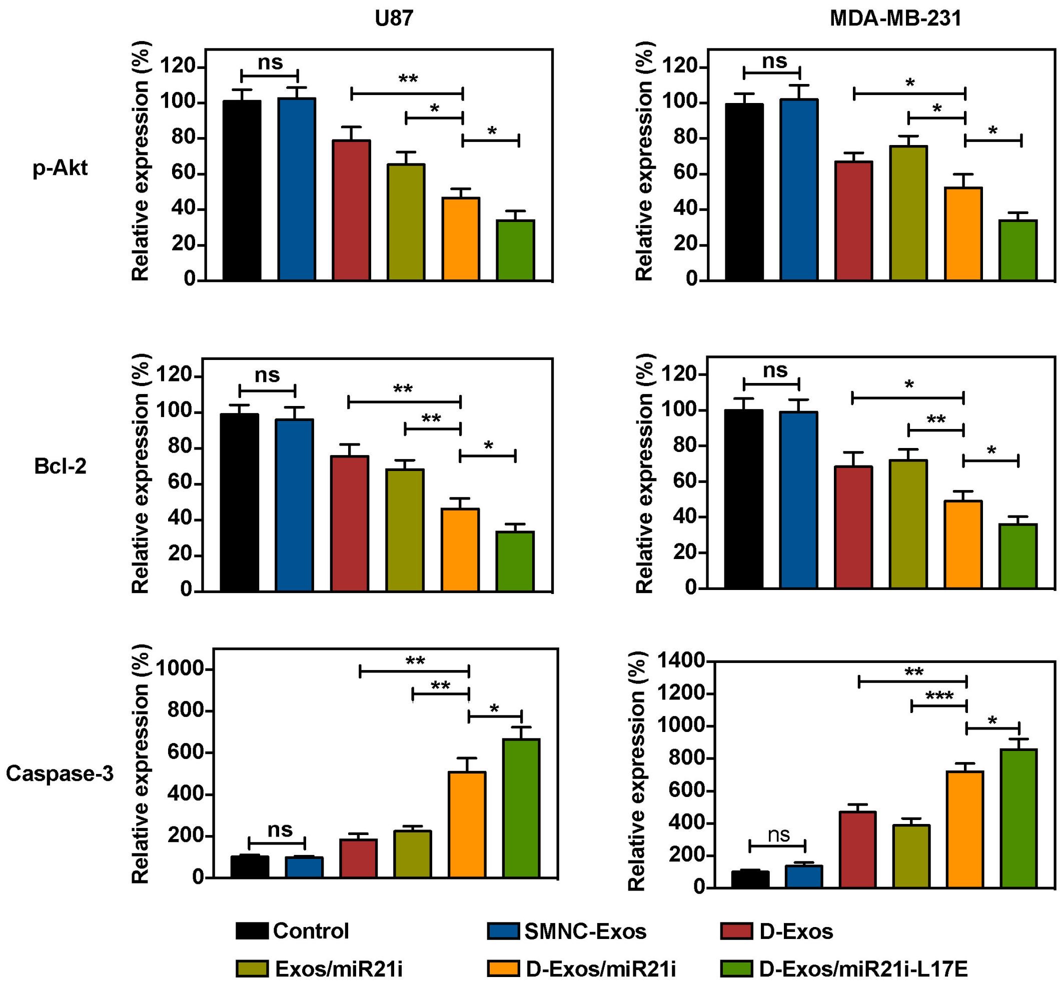

Figure S12. Quantification of the protein expression level is shown by normalized values. Data are shown as the mean ± SD (n=3). Significance levels are shown as ns $p > 0.05$, * $p < 0.05$, ** $p < 0.01$, *** $p < 0.001$.

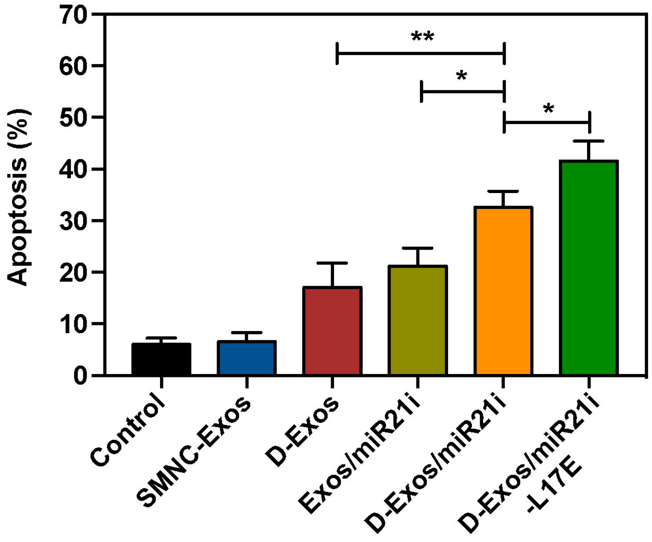

Figure S13. Apoptosis percentage of cells with different treatments. Data are shown as the mean ± SD (n=3). The significant levels are shown as * p < 0.05, ** p < 0.01.

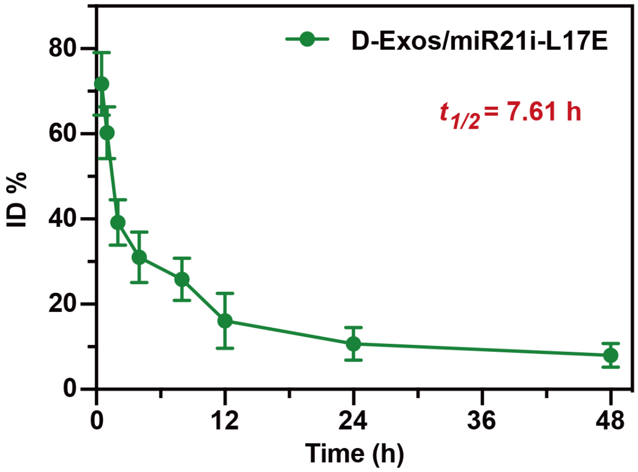

Figure S14. Blood circulation lifetime of the D-Exos/miR21i-L17E in U87 tumor-bearing mice after intravenous injection. Data are shown as the mean ± SD (n=5).

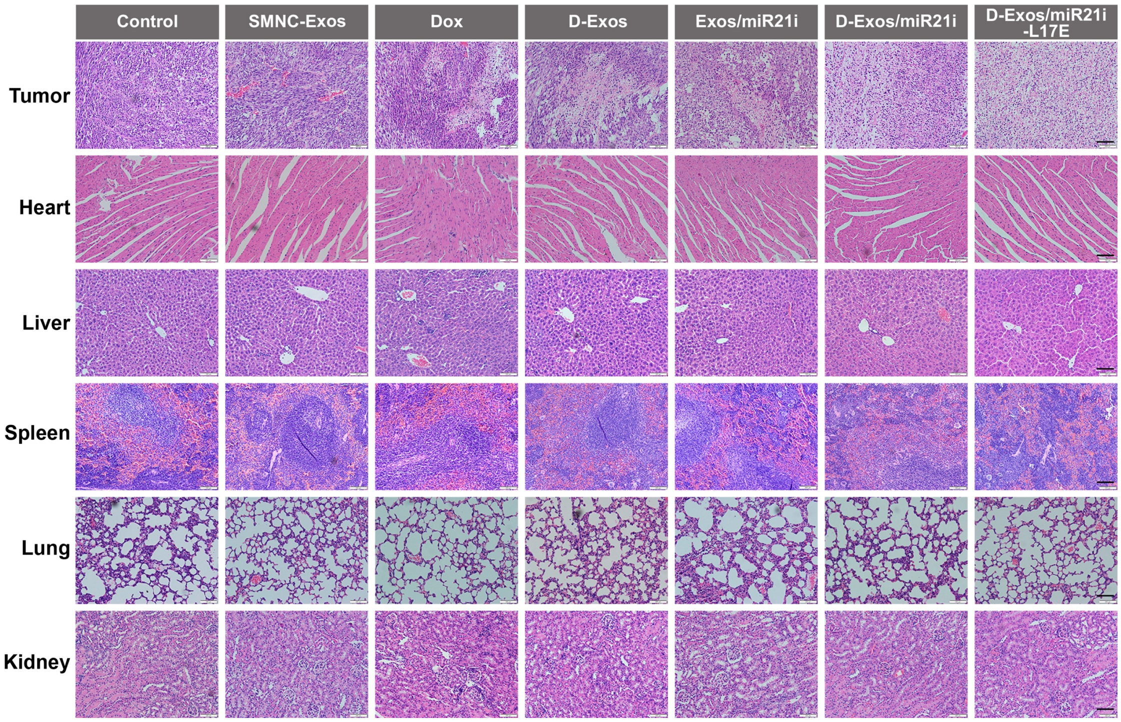

Figure S15. Representative H&E-stained images of tumors and major organs collected from U87 tumor-bearing mice after 18 days of different treatment.

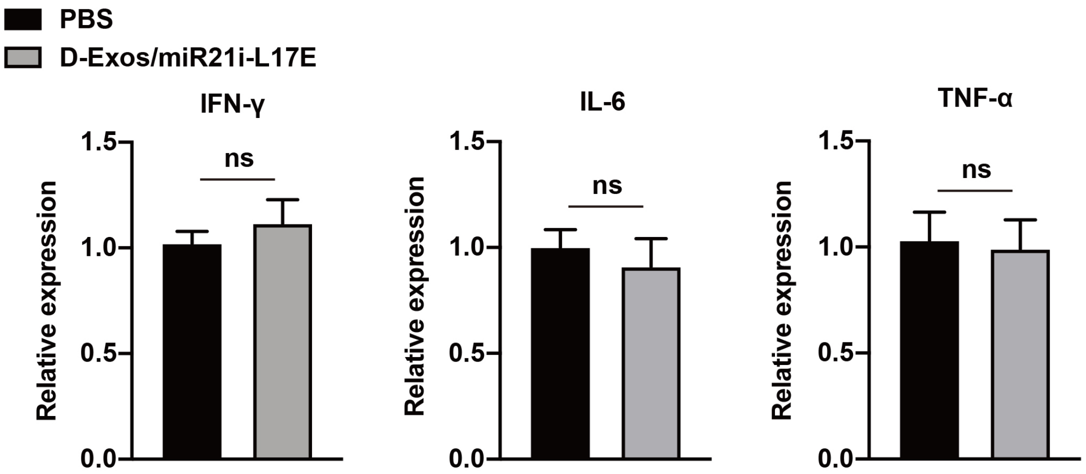

Figure S16. IFN-γ, IL-6, TNF-α levels in serum of KunMing mice after intravenous injection of D-Exos/miR21i-L17E. PBS was used as control. Data represent mean ± SD (n=5).
